# Automated localization and quality control of the aorta in cine CMR can significantly accelerate processing of the UK Biobank population data

**DOI:** 10.1371/journal.pone.0212272

**Published:** 2019-02-14

**Authors:** Luca Biasiolli, Evan Hann, Elena Lukaschuk, Valentina Carapella, Jose M. Paiva, Nay Aung, Jennifer J. Rayner, Konrad Werys, Kenneth Fung, Henrike Puchta, Mihir M. Sanghvi, Niall O. Moon, Ross J. Thomson, Katharine E. Thomas, Matthew D. Robson, Vicente Grau, Steffen E. Petersen, Stefan Neubauer, Stefan K. Piechnik

**Affiliations:** 1 Oxford Centre for Clinical Magnetic Resonance Research (OCMR), Division of Cardiovascular Medicine, Radcliffe Department of Medicine, University of Oxford, Oxford, United Kingdom; 2 William Harvey Research Institute, NIHR Barts Biomedical Research Centre, Queen Mary University of London, London, United Kingdom; 3 Institute of Biomedical Engineering, Department of Engineering Science, University of Oxford, Oxford, United Kingdom; Universita degli Studi di Padova, ITALY

## Abstract

**Introduction:**

Aortic distensibility can be calculated using semi-automated methods to segment the aortic lumen on cine CMR (Cardiovascular Magnetic Resonance) images. However, these methods require visual quality control and manual localization of the region of interest (ROI) of ascending (AA) and proximal descending (PDA) aorta, which limit the analysis in large-scale population-based studies. Using 5100 scans from UK Biobank, this study sought to develop and validate a fully automated method to 1) detect and locate the ROIs of AA and PDA, and 2) provide a quality control mechanism.

**Methods:**

The automated AA and PDA detection-localization algorithm followed these steps: 1) foreground segmentation; 2) detection of candidate ROIs by Circular Hough Transform (CHT); 3) spatial, histogram and shape feature extraction for candidate ROIs; 4) AA and PDA detection using Random Forest (RF); 5) quality control based on RF detection probability. To provide the ground truth, overall image quality (IQ = 0–3 from poor to good) and aortic locations were visually assessed by 13 observers. The automated algorithm was trained on 1200 scans and Dice Similarity Coefficient (DSC) was used to calculate the agreement between ground truth and automatically detected ROIs.

**Results:**

The automated algorithm was tested on 3900 scans. Detection accuracy was 99.4% for AA and 99.8% for PDA. Aorta localization showed excellent agreement with the ground truth, with DSC ≥ 0.9 in 94.8% of AA (DSC = 0.97 ± 0.04) and 99.5% of PDA cases (DSC = 0.98 ± 0.03). AA×PDA detection probabilities could discriminate scans with IQ ≥ 1 from those severely corrupted by artefacts (AUC = 90.6%). If scans with detection probability < 0.75 were excluded (350 scans), the algorithm was able to correctly detect and localize AA and PDA in all the remaining 3550 scans (100% accuracy).

**Conclusion:**

The proposed method for automated AA and PDA localization was extremely accurate and the automatically derived detection probabilities provided a robust mechanism to detect low quality scans for further human review. Applying the proposed localization and quality control techniques promises at least a ten-fold reduction in human involvement without sacrificing any accuracy.

## Introduction

Aortic distensibility is an independent predictor of cardiovascular morbidity and mortality [1] that can be estimated non-invasively by cine CMR (Cardiovascular Magnetic Resonance) as the change of aortic lumen area from diastole to systole, divided by central pulse pressure. Manual contour tracing is very time consuming and suffers from high inter- and intra-observer variability, so in the last decade semi-automated methods have been developed to assess lumen area more rapidly and reduce variability [2,3]. These methods still require visual quality control of cine series (up to 100 images per scan), manual localization of the region of interest (ROI) of the ascending (AA) and proximal descending (PDA) aorta, and supervision of the resulting lumen contours. The required amount of time and user interaction is therefore a severe limitation in large-scale population-based studies, such as the UK Biobank population cohort (100,000 scans in total). Consequently, the availability of fully automated methods for AA and PDA detection-localization and image quality assessment would be highly desirable.

The challenge of developing a fully automated analysis pipeline can be divided into 4 parts: 1) detection and localization of AA and PDA, 2) image quality assessment, 3) frame-by-frame AA and PDA lumen segmentation, i.e. contouring, and 4) max-systolic and min-diastolic lumen area estimation. Our study focused on the 1^st^ and 2^nd^ processing steps of the analysis pipeline. Previous studies have applied the Circular Hough Transform (CHT) to localize carotid arteries or aorta in black-blood or phase-contrast CMR [4–7]. However, those studies used small datasets for method validation, some from healthy subjects and mostly without significant artefacts (not real-world data). Additionally, to select the correct ROI for the aorta or carotid arteries from all the candidate ROIs detected by CHT (e.g. to distinguish AA from PDA, pulmonary artery and vena cava), those methods relied on a set of rules based on heuristics and determined on small datasets. Finally, in alternative to the multi-step approach described above, a very recent work has proposed to segment the aortic lumen directly using Recurrent Neural Networks [8].

Our study took a different approach that is data-driven and based on state-of-the-art Computer Vision and Machine Learning techniques, to leverage the unprecedented amount of data available in the UK Biobank cohort. Contrary to previous studies, we propose to learn the characteristics of AA and PDA from cine data, using feature extraction techniques to capture and describe information about the local CMR characteristics in the ROI. The purpose of this study was to develop a fully automated method to 1) detect and locate the ROIs of AA and PDA, 2) provide a mechanism to detect low quality scans, and to assess its performance on a large dataset from the UK Biobank population.

## Materials and methods

### Dataset

The dataset comprised the first 5100 aortic cine scans in the UK Biobank cohort from 4996 subjects (repeated scans per subject were included) imaged on a 1.5 Tesla scanner (Siemens Aera, Syngo Platform VD13A) in a single centre (Cheadle, UK). Retrospectively ECG-gated cine images were acquired using a transverse balanced Steady State Free Precession (bSSFP) sequence at the level of the pulmonary trunk and right pulmonary artery during breath-hold. Typical acquisition parameters were TE = 1.17 ms, TR = 2.8 ms, flip angle = 60°, Grappa factor = 2, acquired matrix size = 240×196, voxel size = 1.6×1.6×6 mm^3^, and actual temporal resolution = 28 ms, which was interpolated to 100 images per cardiac cycle with resolution ~10 ms [9]. The study has been performed on fully anonymised data, under the ethics governed by the UK Biobank. Written consent was gained from all study subjects. For further details see http://www.ukbiobank.ac.uk/ethics.

### Ground truth

Ground truth (GT) data were manually generated by 13 human observers across 2 core imaging laboratories in Oxford and London. The observers had different backgrounds and levels of CMR experience, ranging from 1 to 15 years (average 4.3 years). 6 of them had a technical background in image analysis, and 7 were medical doctors training in cardiology. Observers assessed the overall image quality (IQ) of the cine scans and assessed the location and radius of AA and PDA, following the instructions detailed in the Standard Operating Procedures (SOP, S1 File). Each observer was trained on a randomized subset of 30 scans that included reference examples of different artefacts and degrees of quality. Training results for ROI localization and IQ scores were individually reviewed with LB (12 years of experience in CMR Physics and Image Analysis). Each observer analysed approximately 800 scans (randomly assigned) and repeated the analysis on 100 scans to calculate intra-observer variability. Manual analysis was performed using a dedicated Matlab (MathWorks) interface developed for this purpose, which loaded the randomised scans and presented them sequentially to streamline the process and minimize the time required.

**Fig 1:**
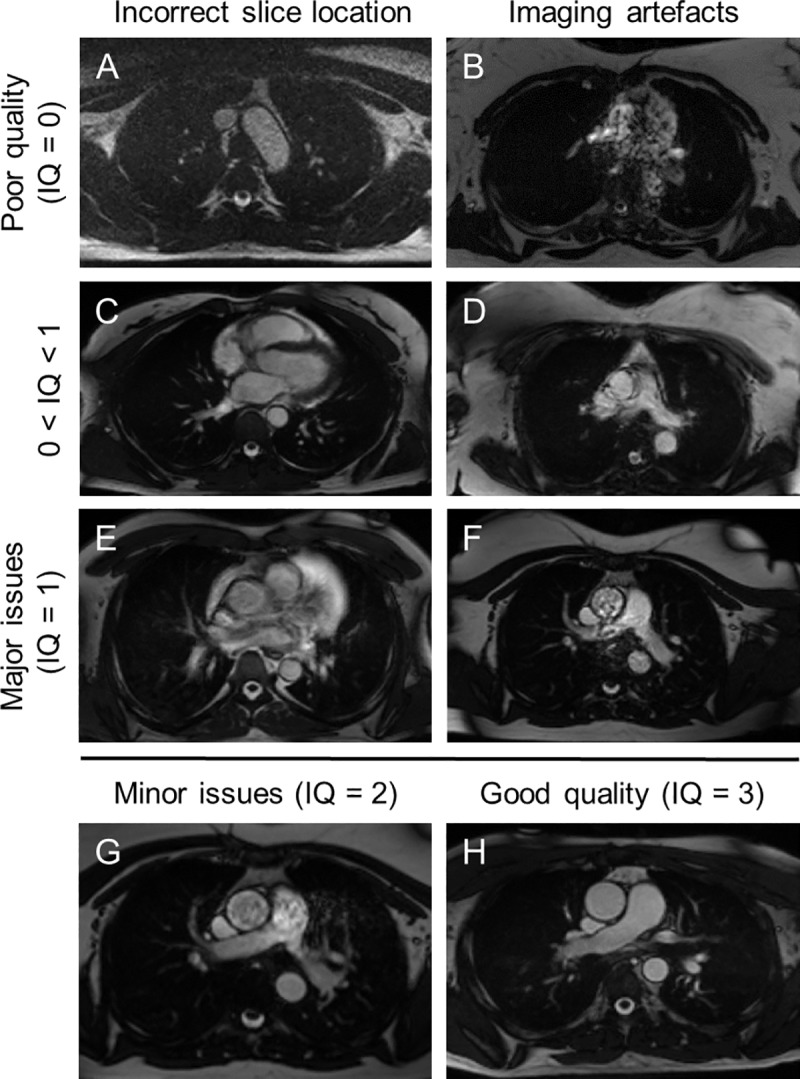
Example images with different image quality (IQ) scores. Poor quality (IQ = 0) due to incorrect slice location (A) and extreme imaging artefacts (B). 0 < IQ < 1 due to incorrect slice location (C) and severe artefacts (D). Major issues (IQ = 1) due to slice location (E) and artefacts (F). Example images with (G) minor issues (IQ = 2) and (H) good quality (IQ = 3). Please refer to the methods section in the main text for their definitions.

In the 1st window (S1 Fig), the application interface showed the cine images acquired during the cardiac cycle and asked to score the overall cine IQ selecting one of the following categories:

- **Poor quality** (IQ = 0) if the slice location was completely incorrect and both AA and PDA were not visible (example in Fig 1A) or if the images were affected by severe artefacts and both AA and PDA lumen boundaries were corrupted throughout the cardiac cycle (example in Fig 1B).
- **Major issues** (IQ = 1) if images were affected by artefacts or if the slice location was incorrect. In this case AA and PDA lumen boundaries could be corrupted, but still be visible for most of the cardiac cycle (example in Fig 1F). This option was also selected if either AA or PDA was not visible (example in Fig 1E).
- **Minor issues** (IQ = 2) if the cine quality was sub-optimal due to slice location or minor artefacts, but AA and PDA lumen boundaries could still be easily and consistently identified (example in Fig 1G).
- **Good quality** (IQ = 3) if the images were acquired at the correct slice location, were artefact-free with sharp AA and PDA lumen boundaries and good image contrast throughout the cardiac cycle (example in Fig 1H).

To provide a reliable IQ score for each scan, the visual assessment followed this procedure:

1. Each scan was validated by 2 independent observers to calculate inter-observer variability, measured by the intra-class correlation coefficient (ICC) using SPSS (IBM).
2. The final IQ score was calculated as the average between the 2 observers weighted by their individual intra-observer ICC.
3. If the difference between IQ scores was > 1 the scan was reviewed by 3 observers (EL, EH and LB, with 15, 1 and 12 years of CMR experience respectively) who scored the IQ by consensus.

Completely manual annotation of 5100 scans was not feasible, so we used the following strategy:

1. One operator annotated the ROIs of AA and PDA on a subset of data (200 scans), which were used to train the automated algorithm (described in the next sections) and provide initial predictions for AA and PDA on all scans.
2. The initial ROI annotations were assessed and corrected by 2 independent observers on the 2^nd^ window (S2 Fig) of the application interface. AA or/and PDA could also be categorised as ‘not visible’ due to severe artefacts or incorrect slice location.
3. The results of the 2 observers were averaged or, in case of substantial disagreement, reviewed by a 3rd observer. This final step generated the GT annotations.

At the end of this 3-stage process, all 5100 scans had GT data that were used to train and test the automated detection algorithm. The Dice Similarity Coefficient (DSC) was used to calculate the spatial overlap between the GT binary mask and that of the automatically detected ROI:

$$DSC(M_{ROI},M_{GT})=2∙\frac{{|M}_{ROI}\cap M_{GT}|}{\left|M_{ROI}|+{|M}_{GT}\right|}$$

where *M*_*GT*_ and *M*_*ROI*_ were the sets of voxels belonging to the foreground of the corresponding binary masks, and |*M*| indicated the number of elements in the *M* set.

### ROI detection and feature extraction

**Fig 2:**
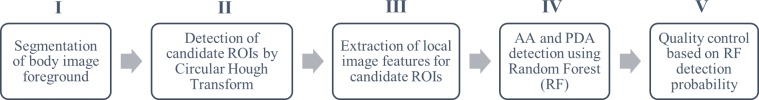
Algorithm flowchart. Automated Ascending and Proximal Descending Aorta (AA and PDA) detection-localization and quality control.

We developed a fully automated algorithm to detect and localize AA and PDA, which was implemented in Matlab using the VLFeat Toolbox [10]. This section describes the detection of candidate ROIs and the extraction of their local features, while the next section is concerned with supervised machine learning. The main processing steps are shown in Fig 2.

**I**) The foreground (i.e. the axial image of the body at the thorax level) was segmented from the background using Otsu’s thresholding method on the Gaussian-filtered image (σ = 5), followed by morphological operations (dilation and erosion) on the largest connected components to close the binary mask (using a circular structuring element of radius = 10), and finally calculate its convex hull. Images and binary masks were then interpolated to 480×392. For different scans, the axial image of the body can vary significantly in size and position within the field of view (FOV). Thus, by determining the location and extent of the foreground, this image processing step helps focusing the search area for candidate ROIs (in step 2) and defining a subject-specific reference frame (in step 3) that is independent of the FOV centre.

**Fig 3:**
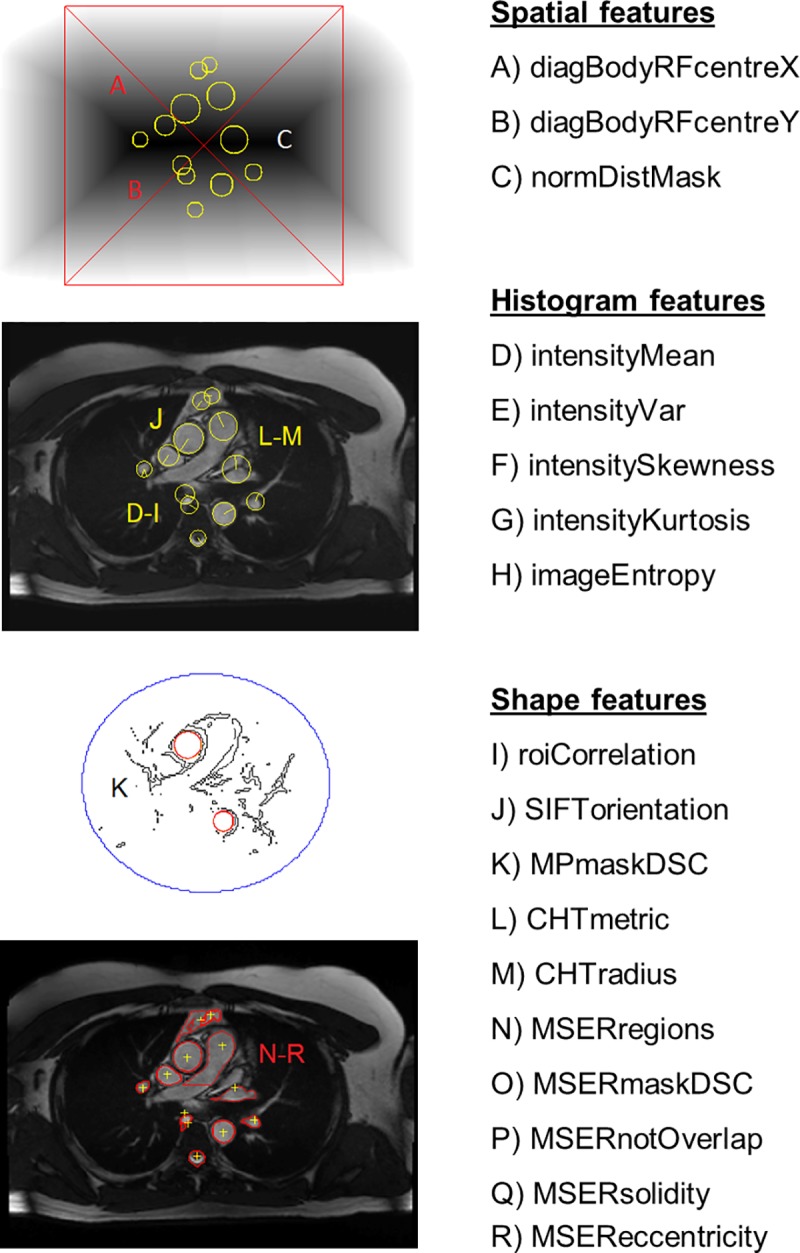
ROI features. Graphical representation and list of local features (A-R) extracted for each candidate ROI (yellow circles) and divided into groups. Spatial features: diagonal axes A-B and distance mask C of the body. Shape features: J is indicated by the line inside the circles; K is represented by the red circles on the Motion Periodicity (MP) map; L and M describe the circles detected by the Circular Hough Transform (CHT); and N-R characterize the Maximally Stable Extremal Regions (MSER) marked by the red contours. Graphical representation was not possible for features D-I. Please refer to the methods section in the main text for the definition of each image feature.

**II**) To find candidate ROIs for AA and PDA, anatomical structures were detected using the Circular Hough Transform (CHT), similarly to what was previously described for carotids [5]. The gradient image was computed by convolution with the Sobel operator and strong edges were detected by Otsu’s thresholding method. Edges cast their votes into a complex accumulator array (480×392), encoding the radius as phase, and producing peaks (with magnitude normalized from 0 to 1) that corresponded to the circle centres. The set of candidate ROIs was selected as the local maxima in the accumulator array with magnitude > 0.1. The algorithm searched for bright-blood lumina with a wide range of radii (6–36 mm) and was sensitive to vessels affected by imaging artefacts or pathologies, which appeared as broader peaks in the accumulator array. The CHT search area was restricted to the core of the body mask (foreground segmented in step 1), automatically defined as the ellipse centred on the intensity-weighted centroid with axes equal to 0.6 and 0.7 times the maximum anterior-posterior size of the body mask (blue ellipse in Fig 3). By using CHT to provide a set of candidate ROIs, this step reduced the complexity of the AA-PDA localization problem compared to other possible approaches (e.g. sliding-window detection, or voxel-based classification).

**III**) For each candidate ROI, the algorithm extracted 18 local features engineered to represent interpretable characteristics of the ROIs, i.e. spatial location, local distribution of the intensity, motion, anatomical size and shape, as described below and visualized in Fig 3.

#### Spatial features

The body mask (segmented in step 1) was used to define a new reference frame centred on the intensity-weighted centroid and with axes along the diagonals of a square box with sides equal to the maximum anterior-posterior size of the body mask. **A)** *diagBodyRFcentreX* and **B)** *diagBodyRFcentreY* were the coordinates of the ROI centre in the body reference frame, while **C)** *normDistMask* indicated the distance from the body mask boundary and was calculated as the average value of the normalized Euclidean distance transform of the binary mask.

#### Histogram features

The distribution of ROI intensities was described by **D)** *intensityMean* and the statistical moments **E)** *intensityVar*, **F)** *intensitySkewness* and **G)** *intensityKurtosis*. Information content was measured by average Shannon entropy **H)** *imageEntropy H*(*z*) = −∑_*i*_*p*(*z*_*i*_) *log*_2_(*p*(*z*_*i*_)), where *p*(*z*_*i*_) is the normalized histogram of intensity *z*, with *i* = 0,1,…,*N*−1.

**Shape features** of the anatomical structures were extracted using different techniques. **I)** *roiCorrelation* was the 2D correlation coefficient between the ROI of the original image and the binary mask of the circle detected by CHT. **J)** *SIFTorientation* represented the local orientation (in radians) of the Scale Invariant Feature Transform (SIFT) descriptor [11] calculated at the ROI centre and at scale = *CHTradius*. It was computed as the maximum of the histogram of gradient directions (36 bins covering 2π rad) in a Gaussian-weighted circular window with SD = 1.5∙scale. **K)** *MPmaskDSC* characterized the periodicity of aortic wall motion throughout the cardiac cycle, i.e. aortic wall distension (systole) and recoil (diastole). The temporal frequencies of individual voxels were computed by the Fourier Transform of the time series of cine signal intensities. Voxel spectra were then normalized and thresholded to detect the frequency with highest magnitude that identified periodic motion during the cardiac cycle. This generated a binary image, which was then filtered to detect boundary voxels and obtain the motion periodicity (MP) map. CHT was then applied to the MP map to find circular structures and calculate their DSC against candidate ROIs. **L)** *CHTmetric* indicated the strength of the peak in the CHT accumulation array, ranging from 0 to 1, while **M)** *CHTradius* was the estimated radius of the circular ROI. **N)** *MSERregions* was the number of Maximally Stable Extremal Regions (MSERs) [12] detected in a square centred on the ROI centre and with side = 6∙*CHTradius* (extremal regions are defined as the connected components of the intensity level sets). The step size between intensity threshold levels was set to 1% of the maximum intensity. Among the extremal regions, those that were maximally stable, i.e. their areas changed the least, were selected as MSERs. The maximum area variation between extremal regions at varying intensity thresholds was set to 50%. **O)** *MSERmaskDSC* denoted the maximum overlap measured by DSC between MSERs and the candidate ROI and identified the corresponding MSER as optimal (OMSER) for further analysis. **P)** *MSERnotOverlap* was calculated as ${|M}_{OMSER}\cap M_{ROI}^{C}|/{|M}_{ROI}|$, i.e. the proportion of the OMSER not overlapping with the candidate ROI. **Q)** *MSERsolidity* indicated the convexity of OMSER and was calculated as the ratio between the OMSER area and the area of its convex hull. **R)** *MSEReccentricity* represented the eccentricity of OMSER and was computed from the ellipse with equivalent second-order moments.

Finally, in order to compare detection accuracy, the general approach used to select the vessels of interest in previous studies [4–6] was replicated, i.e. the local maxima search in the Hough domain was constrained by a set of rules based on heuristics and organized in a simple classification tree: AA had to be bigger, anterior and to the right of PDA, and their distance ≥ 18 mm [6].

### Supervised learning with random forest

Once circular structures were detected by CHT as candidate ROIs and local image features were extracted for each of them, the task of identifying AA and PDA among all the candidates (Fig 2-IV) was formalised as a multinomial classification problem, i.e. that of assigning one of the 3 possible classes—AA, PDA, or NA (Not Aorta)—to each ROI. The classification was performed by a supervised learning technique called Random Forest (RF) using GT location and radius of AA and PDA provided by the human observers. The RF algorithm learned the feature statistics of AA and PDA from a training set of labelled scans, in which the circular ROIs scoring DSC > 0.75 against GT were categorised as AA (or PDA), while the others were marked as NA, i.e. identifying other anatomical structures. When building the RF model, prior probabilities for the 3 classes (NA, PDA and AA) were empirically estimated by the algorithm from the class frequencies in the training data.

The RF algorithm is based on bootstrap aggregation (bagging) and random feature selection to build an ensemble classifier from weak learners [13]. RF provides computationally-efficient multinomial classification with confidence estimation, is robust to noisy and correlated features, and can compute the relative importance of features. RF combines the results of an ensemble of classification trees, which are characterized by low bias and high variance. By ensuring low correlation among the individual classification trees, RF minimizes the risk of overfitting the training data and yields a classifier with both low bias and low variance. This was achieved by randomizing the process of building the forest.

Different bootstrap samples—randomly selected with replacement from observations in the training set—were used to train different trees, the results of which were aggregated into the RF model. This procedure used a bag containing 63.2% of the observations to build the trees in the forest. The out-of-bag (OOB) observations were not in the training set of each tree, so they were used as a validation set to estimate the RF performance internally. The size of the training dataset and the optimal internal parameters for our RF model (the number of randomly selected features per decision split and the number of trees) were estimated using OOB observations. The relative feature importance was derived from OOB permutations, i.e. the contribution of a feature to the RF performance was estimated from the increase in classification error due to the values of that feature being randomly permuted across the OOB observations.

A fixed-size subset of the available features was randomly sampled at each node (decision split) in each classification tree. From the subset, the algorithm split the tree using the feature that maximized the decrease of node impurity Δ*I*_*n*_, as measured by the Gini index $I_{n}=\sum_{c=1}^{3}f_{c}(1-f_{c})$ where *f*_*c*_ represented the fraction of observations at node *n* that belong to class *c* = 1 (NA), *c* = 2 (PDA), and *c* = 3 (AA). The decrease of Gini impurity obtained by splitting the parent node *n* into child nodes *l* and *r* is given by Δ*I*_*n*_ = *I*_*n*_−*f*_*l*_*I*_*l*_−*f*_*r*_*I*_*r*_ where *f*_*l*_ and *f*_*r*_ are the sample fractions from node *n* to node *l* and *r*. Each individual tree in the RF was grown to its full depth (unpruned). A termination node (leaf) of a tree was reached when there was no splitting that could decrease the Gini impurity (Δ*I*_*n*_ = 0) and the observation was assigned to the most probable class.

In the test dataset, the features extracted for each candidate ROI of every scan were passed down the trees in the RF model to calculate the probability of belonging to class AA, PDA, or NA. Class posterior probabilities were determined by the average over all the trees in the RF model, and the predicted class for an ROI was the class with maximum posterior probability. In every scan there could be more than one candidate ROI identifying AA (or PDA) with slightly different location and radius. Hence, only the best predictions, i.e. the ROIs with maximum AA and PDA probabilities, were selected.

Finally, we hypothesized that these probabilities could be used as an automated mechanism for quality control (Fig 2-V). To investigate the association between the combined AA×PDA detection probabilities and the IQ scored by human observers for the scans in the test dataset, we performed the Kruskal-Wallis rank test and multiple pairwise comparisons using the Bonferroni method at 5% confidence level. We then divided the scans into 2 groups using different IQ cut-offs, performed the Mann-Whitney-Wilcoxon test, and analysed the Receiver Operating Characteristic (ROC) curves to identify an optimal threshold for AA×PDA detection probability that can be used for quality control.

## Results

### Ground truth

Each observer scored the IQ of 800 scans and repeated on 100 of the 800 scans. Single measures of intra-observer consistency were good (median ICC = 0.65, IQR = 0.62–0.71, range = 0.47–0.85). Inter-observer ICC was calculated in 39 randomly allocated combinations of 100 repeat cases. Absolute agreement was fair/good (median ICC = 0.59, IQR = 0.52–0.63, range = 0.27–0.71), while consistency was better (median ICC = 0.74, IQR = 0.68–0.77, range = 0.42–0.83), indicating the presence of small systematic differences between observers. IQ difference between observers was > 1 in 342 out of 5100 scans that were reviewed by a panel to assign a final IQ by consensus. Final IQ scores were calculated as the average between 2 independent observers weighted by their individual intra-observer ICC. The distribution of IQ scores (example images in Fig 1) was the following:

- 94 scans (1.8%) were classified as poor quality (IQ = 0);
- 116 (2.3%) had weighted IQ > 0 and < 1;
- 462 (9.1%) had major issues (IQ = 1);
- 651 (12.8%) had weighted IQ > 1 and < 2;
- 1062 (20.8%) had minor artefacts (IQ = 2);
- 1717 (33.7%) had weighted IQ > 2 and < 3;
- 998 (19.6%) were found to be of good quality (IQ = 3).

AA and PDA location and radius in 5100 scans were validated by 2 independent observers, who disagreed substantially on AA in 45 scans and on PDA in 33 scans, which were reviewed by a 3^rd^ observer. Both AA and PDA were not visible in 26 scans (observers could not reliably identify the location and draw the lumen boundary) due to very poor image quality (average IQ = 0.1) and incorrect slice location (in 9 cases), as exemplified in Fig 1A and 1B. In other 63 scans, only AA was not visible due to poor image quality (average IQ = 0.5) and incorrect slice location (in 54 cases), as exemplified in Fig 1C and 1D. In other 4 scans, only PDA was not visible due to very poor image quality (average IQ = 0). In total, AA was not visible in 1.8% and PDA in 0.6% of the 5100 scans. In all the other scans, the validated locations and radii of AA and PDA were used to generate binary masks representing the GT data.

### Algorithm training: Optimization

**Fig 4:**
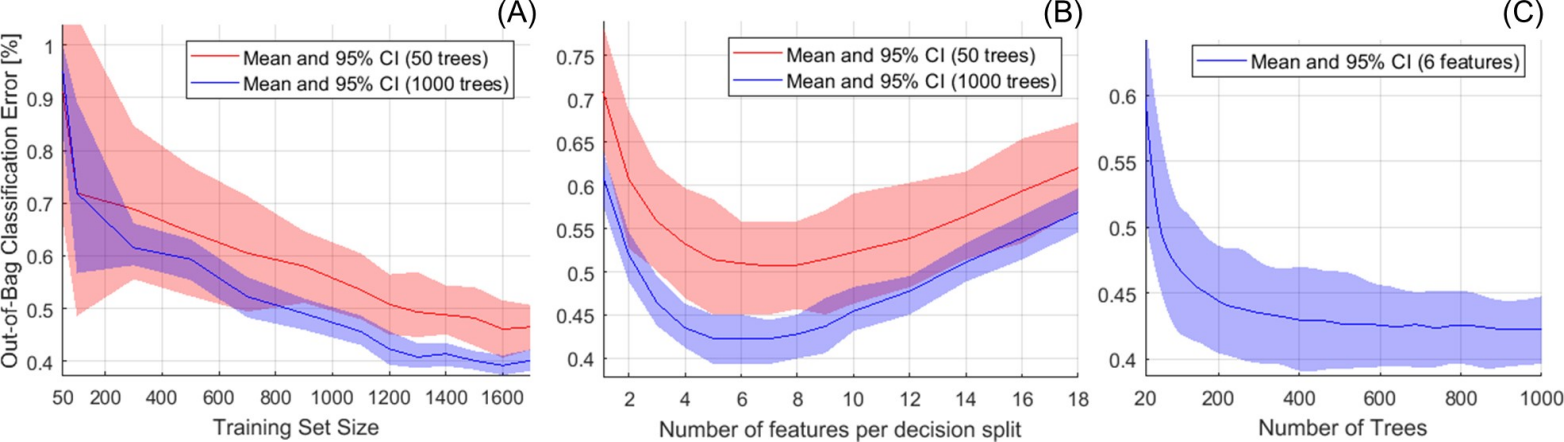
Optimization of the random forest (RF) parameters. Out-of-bag (OOB) mean classification error and CI were calculated from RF training repetitions. A) Training set size for RF with 50 and 1000 trees. B) Number of features per decision split for RF with 50 and 1000 trees. C) Number of trees for RF with 6 features per split. Total number of scans in the training dataset = 1200.

The most important parameters of the RF model were optimized using the OOB observations of the training dataset (Fig 4). Mean OOB error and 95% confidence intervals (CI) were calculated from bootstrapped repetitions of the RF training. Increasing the training set size reduced the mean OOB classification error and CIs up to 1200 scans, where the error stabilized around 0.4% for 1000 trees (Fig 4A). Minimum error = 0.42% (CI = 0.39–0.45%) was achieved for 5 to 7 features per decision split (Fig 4B), while adding more trees produced lower error and tighter CIs, as expected (Fig 4C). We thus decided to use the first 1200 of the 5100 scans available to train a RF model with 6 features per decision split and 1000 trees.

In the training dataset, AA was not visible in 30 scans and PDA in 13 (negatives). CHT detected the location and radius of 15757 candidate ROIs (median number of ROIs per scan = 13, IQR = 11–15, range = 5–34), including concentric and overlapping ROIs. The binary masks of the circular ROIs scoring DSC > 0.75 with the GT data were labelled as AA (or PDA), while the others were labelled as NA. In total, the training dataset had 15757 observations, with 12851 ROIs labelled as NA, 1195 as PDA and 1711 as AA. The number of ROIs was higher than the number of AA (or PDA) positives because multiple ROIs could satisfy the criterion DSC > 0.75 with slightly different location and radius.

### Algorithm training: Image features

**Fig 5:**
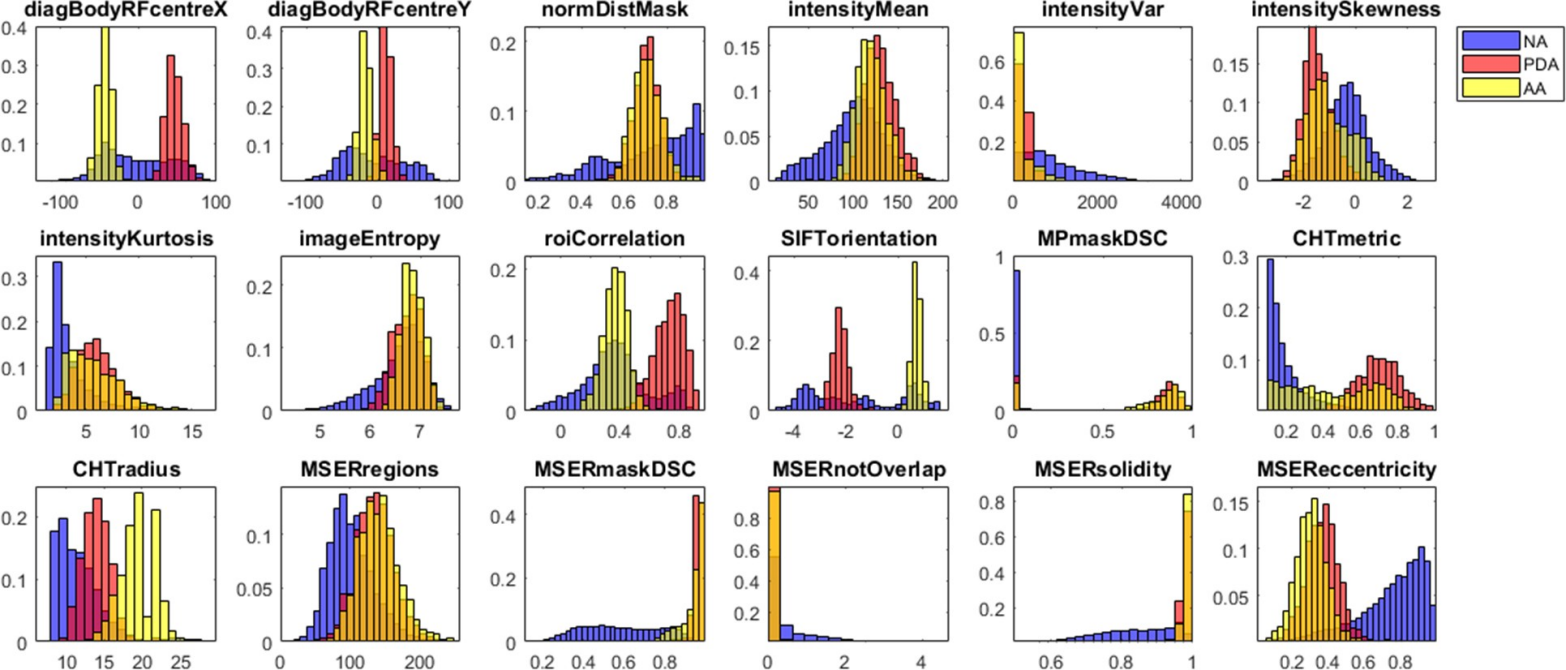
Histograms of image features. ROIs classified as Ascending Aorta (AA), Proximal Descending Aorta (PDA) or Not Aorta (NA). Total number of scans in the training dataset = 1200.

**Fig 6:**
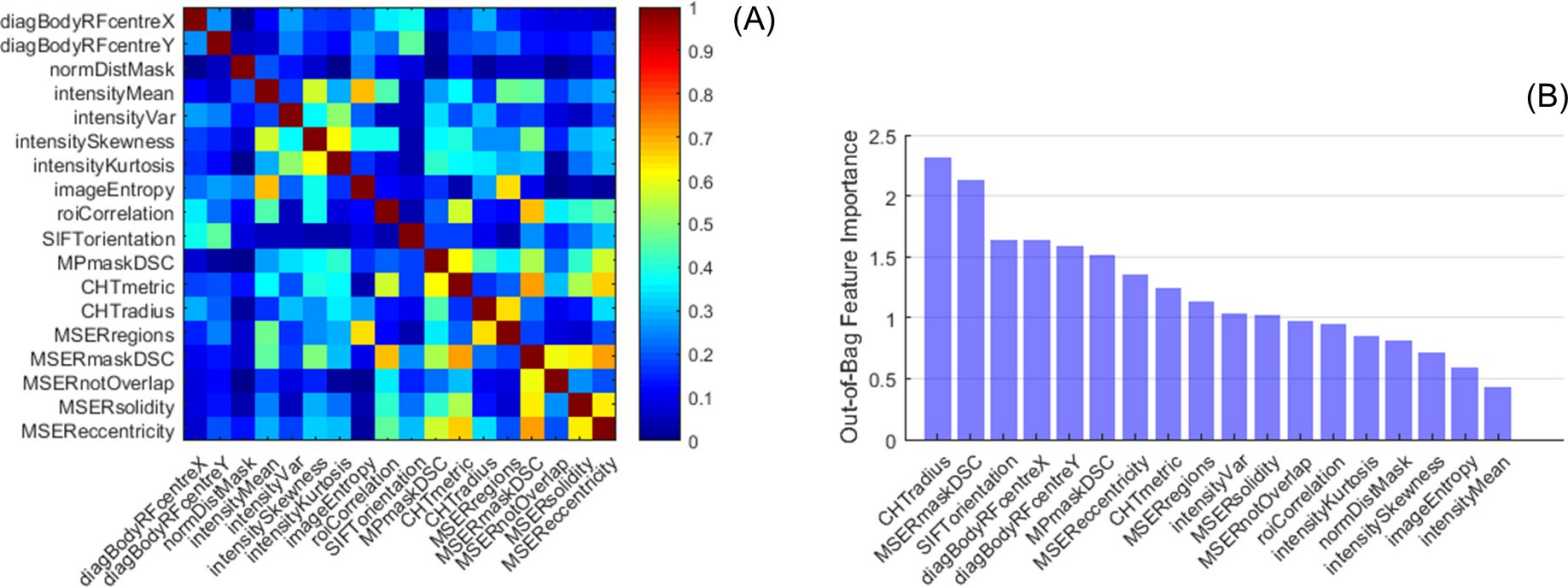
**Absolute correlation between pairs of image features (A) and relative importance (B).** Calculated from out-of-bag (OOB) observations in the training dataset (1200 scans in total).

The probability distributions for the 18 features extracted from the candidate ROIs in the training dataset are shown in Fig 5 for visual assessment. Feature values for ROIs labelled as NA were more heterogeneous (spread-out and multimodal) compared to AA and PDA distributions, which tended to be tighter and unimodal. Some features produced NA, PDA and AA distributions that were similar and overlapping (e.g. *imageEntropy*), hence they were not very informative. For other features, the NA distribution was different from AA and PDA (e.g. *MSEReccentricity*), indicating a good ability to distinguish the aorta from other anatomical structures. Finally, another group of features captured differences between AA and PDA and their distributions were clearly separated (e.g. *SIFTorientation*). The absolute correlation between pairs of features was |R| ≤ 0.7, indicating that they described different local properties and could all potentially contribute to the correct ROI classification (Fig 6A). As expected, maximum correlation was achieved for features belonging to the same group, e.g. *intensityMean* vs. *imageEntropy* for histogram features and *MSERmaskDSC* vs. *CHTmetric*, *MSEReccentricity* or *roiCorrelation* for shape features.

Relative feature importance was estimated by permuting OOB observations (Fig 6B). Overall, shape and spatial features were more discriminant than histogram features for the classification of candidate ROIs. The 2 most important features were *CHTradius*, which showed 3 distinct class distributions, and *MSERmaskDSC*, which was good at differentiating AA and PDA from NA (Fig 5). Other 2 strong shape features were *SIFTorientation* and *MPmaskDSC*. The importance of *MSEReccentricity* and *CHTmetric* could have been reduced by their correlation with *MSERmaskDSC*, which was better at capturing and characterizing the anatomy. The X and Y coordinates of the ROI centre in the reference frame of the body (*diagBodyRFcentreX-Y*) were better predictors than the normalized distance (*normDistMask*). Finally, the most discriminant among histogram features was the ROI variance (*intensityVar*), whereas *imageEntropy* and *intensityMean* did not provide much information. In fact, excluding the 5 least important features in Fig 6B from the RF model had just a small effect on the OOB error, which increased from 0.42% (CI = 0.39–0.45%) to 0.46% (CI = 0.43–0.48%).

### Algorithm testing: Automated aorta localization

The test dataset included 3900 cine scans with GT location and radius for AA and PDA. AA was not visible in 59 scans and PDA in 17 (negatives). A total of 51482 candidate ROIs were detected by CHT (median number per scan = 13, IQR = 11–15, range = 4–31). By replicating the general rule-based approach used in previous studies [4–6], i.e. searching local maxima in the CHT accumulation array subject to constraints, the classification test error was 8.6%. The proposed RF algorithm reduced the error to 0.44% by estimating the ROI probability to belong to class NA, PDA, or AA, based on its features, and assigning it to the most probable class. Table 1 shows the confusion matrix of the RF predictions vs. the actual classes, with mean classification error = 0.44% (CI = 0.41% - 0.45%) estimated from bootstrapped repetitions of the RF training. Notably, most misclassifications were False Negatives for AA and PDA.

**Table 1:** Confusion matrix for predicted and actual ROI classes. Mean classification error = 0.44% (CI = 0.41–0.45%) estimated from RF training repetitions on the test dataset (51482 ROIs).

		Actual Class		
		NA	PDA	AA
**Predicted Class**	NA	41892	87	98
	PDA	1	3850	0
	AA	37	1	5516

**Fig 7:**
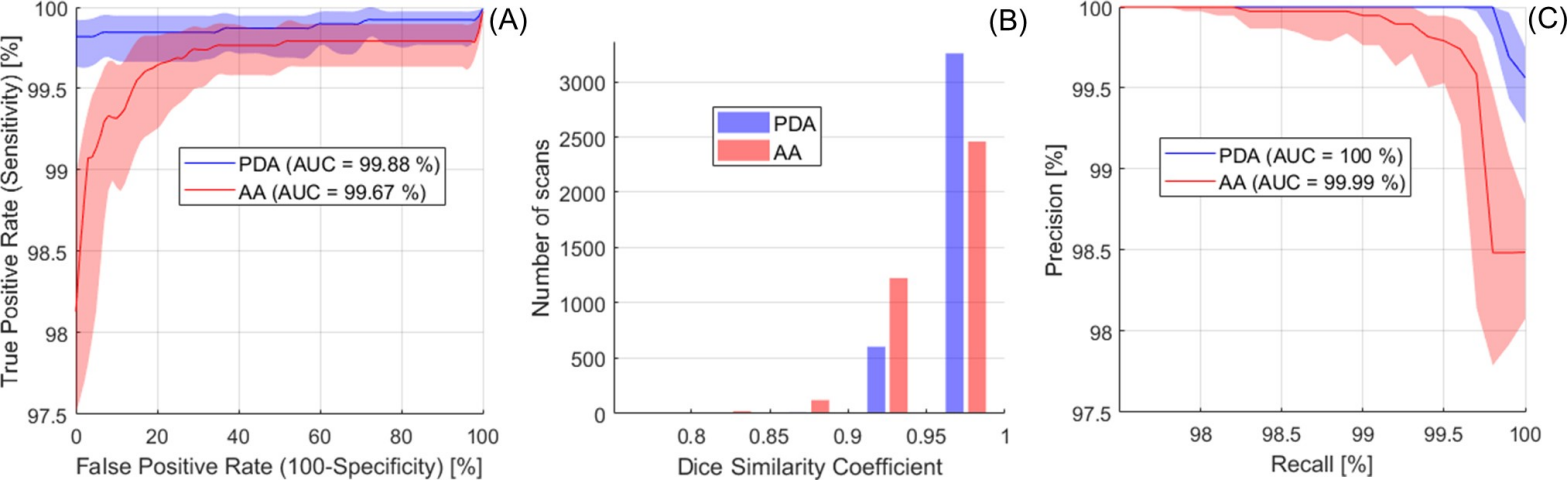
**A) Receiver Operating Characteristic (ROC) and C) Precision-Recall (PR) analysis for AA and PDA detection-localization.** Mean curves and 95% CIs were obtained from bootstrap replicas. Precision baseline was 98.5% for AA and 99.6% for PDA. B) Dice Similarity Coefficient (DSC) for AA and PDA True Positives was ≥ 0.9 in 94.8% and 99.5% of cases, respectively. Total number of scans in the test dataset = 3900.

**Fig 8:**
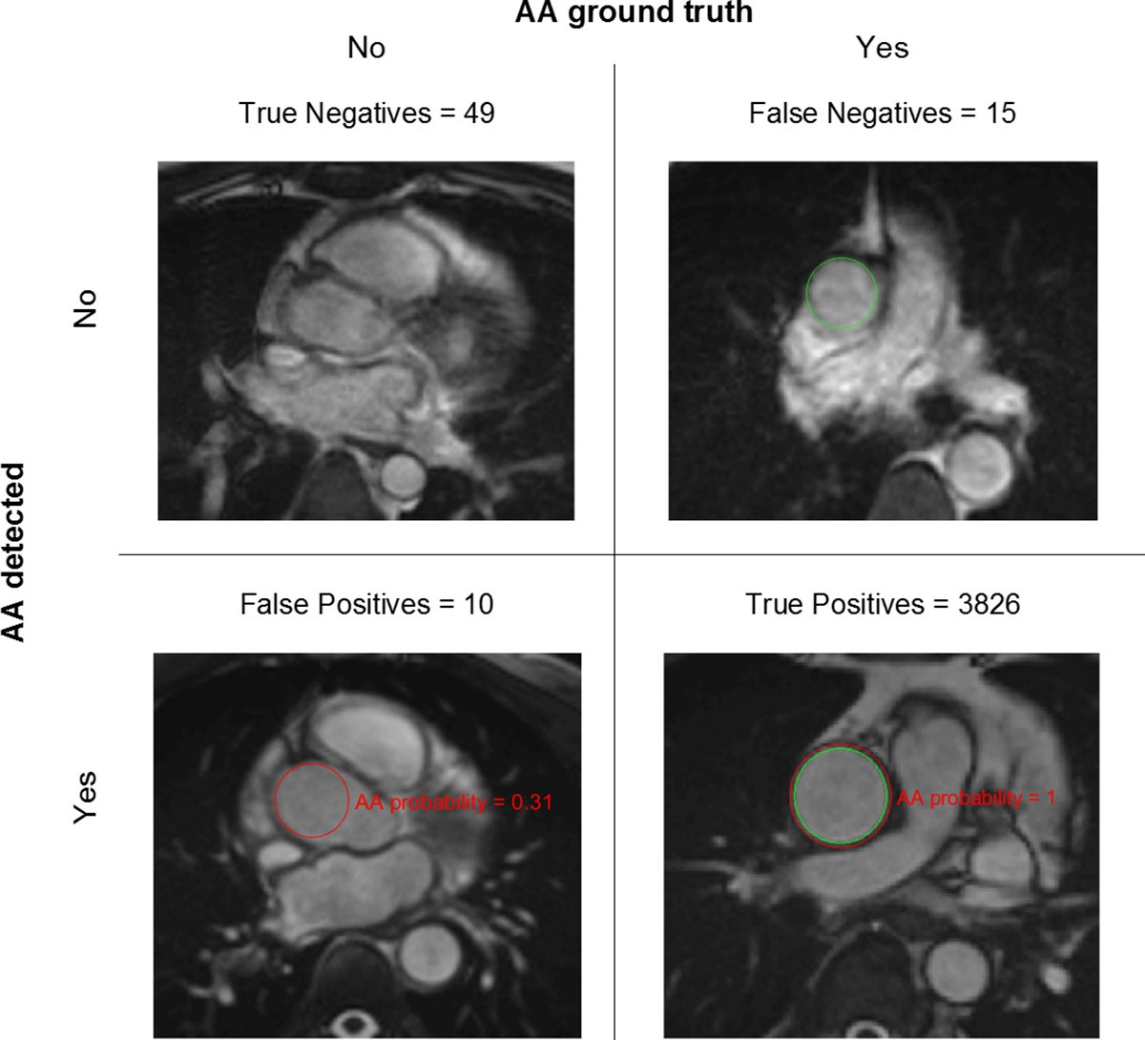
Confusion matrix for AA detection (with example images). Mean error = 0.64% (CI = 0.54–0.74%) estimated from RF training repetitions. Green circles represent the ground truth and red circles the automatically detected ROIs. Total number of scans in the test dataset = 3900.

**Fig 9:**
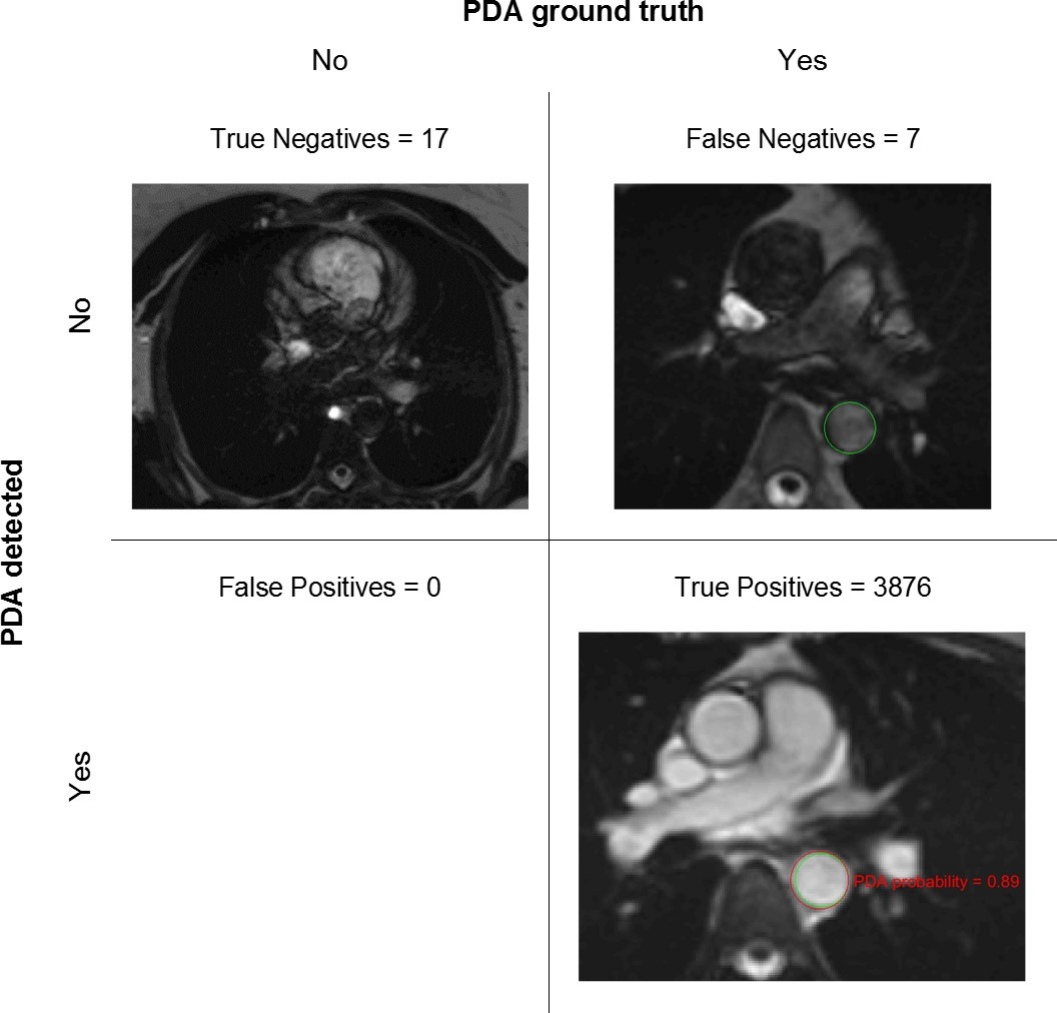
Confusion matrix for PDA detection (with example images). Mean error = 0.18% (CI = 0.15–0.26%) estimated from RF training repetitions. Green circles represent the ground truth and red circles the automatically detected ROIs. Total number of scans in the test dataset = 3900.

The algorithm performance for AA and PDA detection in 3900 scans was illustrated by the Receiver Operating Characteristic (ROC) and Precision-Recall (PR) curves in Fig 7A–7C, where Precision = TP/(TP+FP), with TP = True Positives and FP = False Positives, and Recall = Sensitivity. Area under the curve (AUC) for AA and PDA was close to 100% in both ROC and PR plots, indicating excellent performance (near-perfect for PDA). Optimal probability threshold was 0.18 for AA (sensitivity = 99.6%, specificity = 83%, precision = 99.7%, Youden’s J = 0.826, F_1_ score = 0.997) and 0.09 for PDA (sensitivity = 99.8%, specificity = 100%, precision = 100%, Youden’s J = 0.998, F_1_ score = 0.999). Fig 8 shows examples for each of the possible AA outcomes in the confusion matrix. Mean error for AA detection was 0.64% (CI = 0.54% - 0.74%). The AA ROI was correctly localized in 3826 scans (TP) and the number of True Negatives (TN) was 49. We found 10 FP (typically due to incorrect slice location, as in the example image) and 15 False Negatives (FN; typically, due to blurring, as in the example image). Fig 9 shows examples for each of the possible PDA outcomes in the confusion matrix. Mean error for PDA detection was 0.18% (CI = 0.15% - 0.26%). The PDA ROI was correctly localized in 3876 scans (TP) and the number of TN was 17. We found no False Positives and 7 FN (typically due to artefacts, as in the example image).

The DSC distribution showed excellent agreement between automatically detected ROIs and GT (Fig 7B). DSC was 0.97 ± 0.04 for AA (3826 TP cases) and 0.98 ± 0.03 for PDA (3876 TP cases). In 94.8% of AA and 99.5% of PDA cases DSC was ≥ 0.9. PDA was detected and localized more accurately than AA, as it was typically less affected by motion artefacts and incorrect slice location. Running on a standard desktop computer (Intel i7 3.4 GHz) without any speed optimization, total computation time per scan for AA and PDA detection was around 5–7 s, with image processing and feature extraction taking 3–5 s (depending on the number of candidate ROIs) and RF prediction taking about 2 s.

### Algorithm testing: Automated quality control

**Fig 10:**
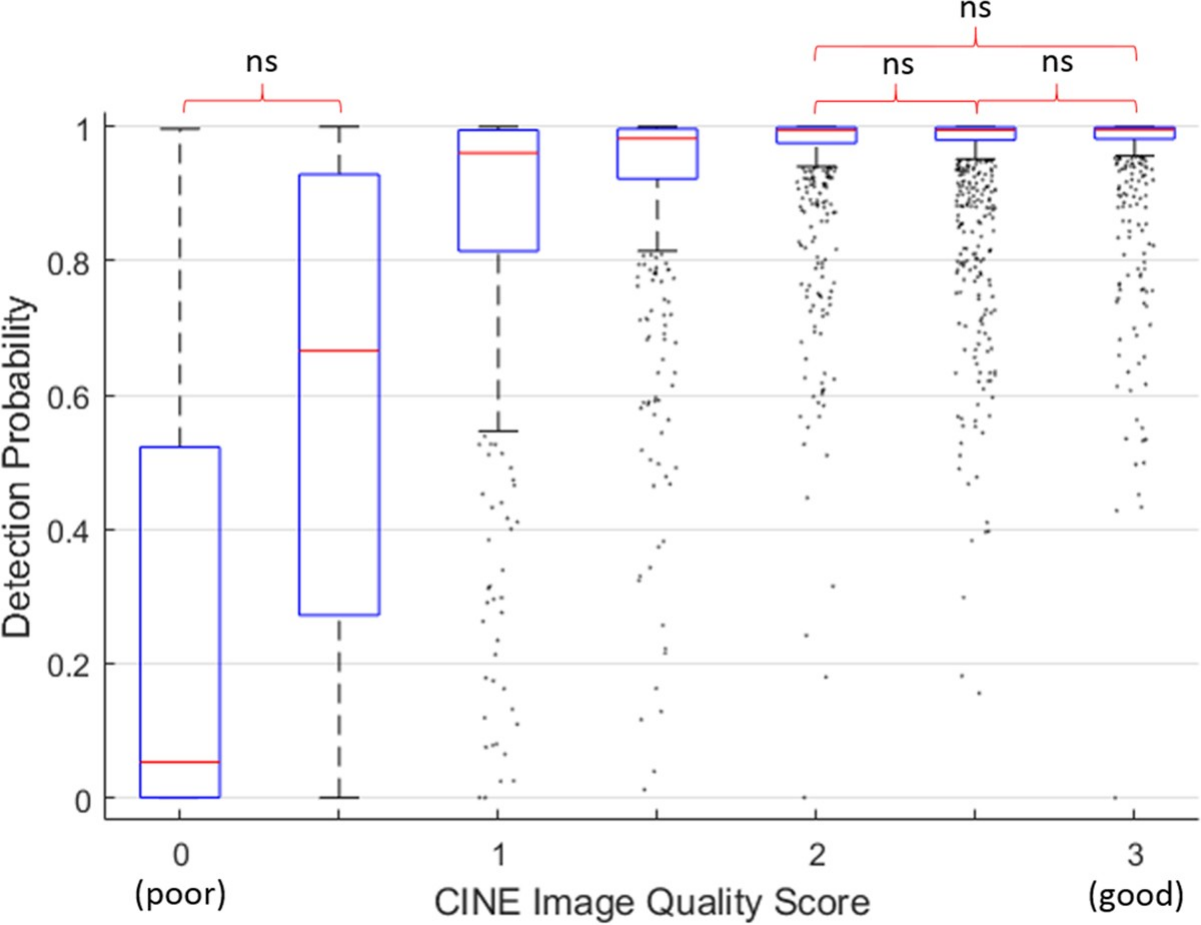
Automated AA×PDA detection probability grouped by CINE image quality (IQ). Total number of scans in the test dataset = 3900. The difference among the highest IQ groups and between the 2 lowest IQ groups is not significant (ns).

In the test dataset (3900 scans), the difference in AA×PDA detection probabilities between groups with high IQ ≥ 2 was not significant, and the same was true for the 2 groups with the lowest IQ, whereas groups with mid-range IQ were significantly different (p < 0.05 using Kruskal-Wallis test with Bonferroni correction) from each other (Fig 10). This showed that detection probabilities were associated with the IQ scores of cine scans and could potentially detect low-quality scans but did not have enough sensitivity to resolve small IQ differences. Hence, to test if detection probabilities could be used to classify low- vs. high-quality scans, we partitioned the scans using 2 possible IQ cut-offs:

- high-quality scans defined by IQ ≥ 1 vs. low-quality scans (IQ < 1), which included only those severely corrupted by artefacts (4% of test data);
- high-quality scans defined by IQ > 1 vs. low-quality scans (IQ ≤ 1), which included also those with major issues (13% of test data).

**Fig 11:**
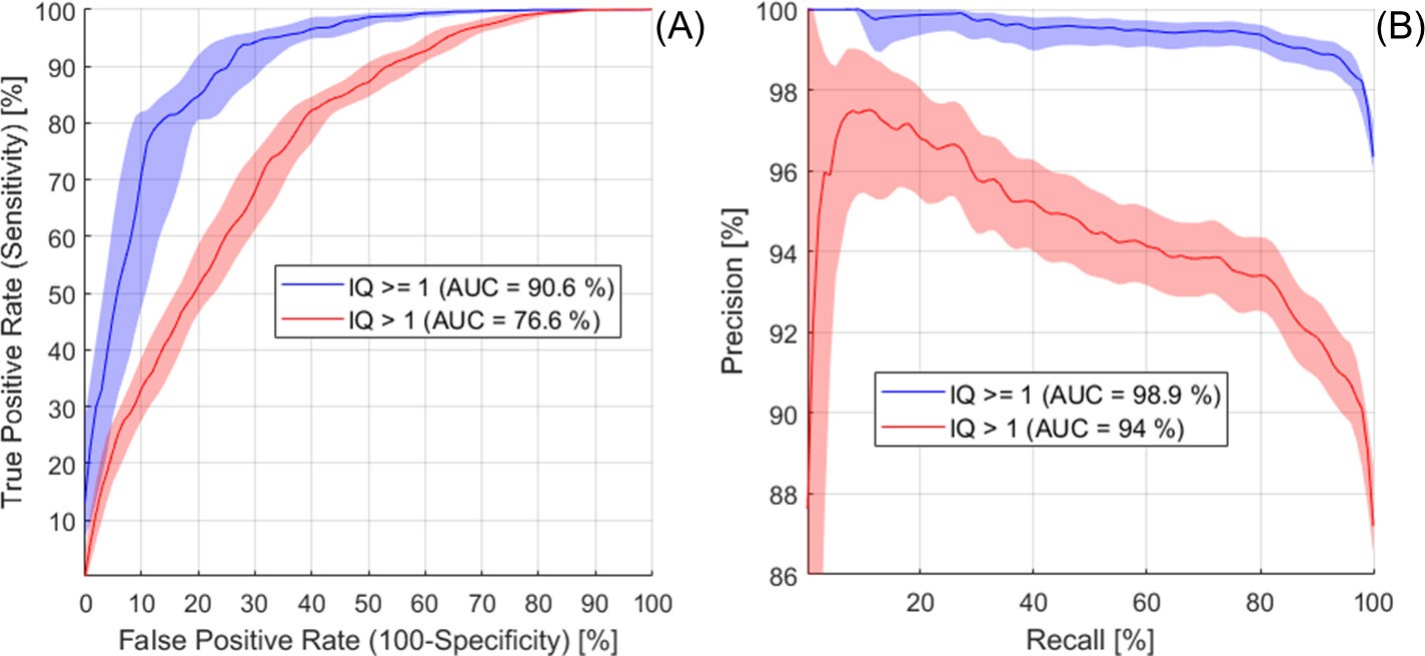
**A) ROC and B) PR analysis for quality control.** Mean curves and CIs (from bootstrap replicas) for 2 possible data partitions (using cut-off at IQ ≥ 1 or at IQ > 1). Precision baseline was 96% for IQ ≥ 1 and 87% for IQ > 1.

The median AA×PDA probabilities of the low- and high-quality groups were significantly different for both IQ cut-offs (p < 0.001 using Mann-Whitney-Wilcoxon test). ROC AUC = 90.6% and PR AUC = 98.9% for partition A (IQ ≥ 1) were both higher than for partition B (Fig 11), indicating that detection probabilities performed better when they were used to discriminate the lowest IQ scans from all the others. ROC and PR curves for partition A showed 2 possible optimal probability thresholds, at 0.75 (sensitivity = 93.8%, specificity = 73%, precision = 98.8%, Youden’s J = 0.67, F_1_ score = 0.96) and 0.95 (sensitivity = 80.7%, specificity = 86%, precision = 99.3%, Youden’s J = 0.67, F_1_ score = 0.89), to identify scans not affected by severe artefacts (IQ ≥ 1).

Using the threshold with the highest F_1_ score (i.e. detection probability < 0.75), 350 out of 3900 scans were identified as low-quality and excluded from the analysis (9% of test dataset). In the remaining 3550 scans the automated detection algorithm was able to correctly localize all the AA and PDA ROIs (100% TP, 100% accuracy). AA×PDA detection probabilities thus offered a method to automatically identify scans that have sufficient IQ, i.e. high probability, and select those below the threshold to be reviewed by human observers.

## Discussion

We developed an automated method to localize the ROIs of AA and PDA and provide a mechanism to detect low quality scans. Using the largest aortic cine CMR dataset to date (5100 scans from UK Biobank), our algorithms were trained on 1200 and tested on 3900 scans, resulting in 99.4% accuracy for AA and 99.8% for PDA. Detection probabilities of images with low- and high-IQ were significantly different and were used as a quality control (QC) method to identify scans severely corrupted by artefacts. In fact, by accepting only the scans with detection probability ≥ 0.75, we obtained 100% accuracy for both AA and PDA detection. These results indicated that in the UK Biobank cohort (100,000 scans when completed) we can expect that less than 10% of scans will have low AA×PDA detection probability and require user interaction, whereas over 90% will have sufficient quality for reliably accurate AA and PDA detection and localization using our automated algorithm.

Previous studies have tried to automate the process of vessel ROI localization by using small datasets (e.g. 8 [5], 10 [7] and 20 subjects [6]) to determine a range of values for the parameters of interest and a set of rules for identifying the vessel ROI, such as 1) limiting the radius range for the vessel search, 2) constraining their relative size, 3) location and 4) minimum distance between them (and additional constraints specific to the image modality). Hard constraints and heuristics determined on a handful of examples are not effective or efficient in real-world situations, as they lack the flexibility to deal with pathologies, different anatomies or sub-optimal image quality. These studies used small datasets to measure detection accuracy. Using multi-slice black-blood images from 20 patients, Biasiolli et al. estimated a detection error = 10.5% for carotid arteries [5]. In a subset of 50 phase-contrast scans, Goel et al. reported an average absolute distance between automatically and manually detected ROIs, i.e. the localization error, of 1.7 ± 1.0 mm for the AA and 0.6 ± 0.7 mm for PDA [6]. Additionally, they reported detection errors ≥ 2% based on an estimate from the outlier analysis. Using multi-slice black-blood images from 10 healthy volunteers, Gao et al. measured localization errors (centre position and radius differences) from 0 to 0.7 mm for the distal descending aorta [7]. Finally, Adame et al. did not report any detection error for the descending aorta in 28 healthy subjects [4]. By replicating the general approach used in previous studies on our test dataset (3900 scans), the detection error was 8.6%. With the help of the feature extraction and RF algorithms presented in this study, the detection error dropped to 0.4% and localization was very accurate for both AA and PDA.

Recently, with the availability of UK Biobank data, there has been an increasing interest on automated IQ assessment and QC for cardiac cine scans, from the analysis of free-text annotations [14], to the detection of missing (apical and basal) slices [15] and of segmentation failure [16]. In this study, we have proposed a different approach that is specific to the aorta and focused on the quality of the ROI, to identify scans corrupted by severe artefacts using the detection probability as QC metric (determined by the set of feature values for AA and PDA). Given the rapid processing, it appears feasible that IQ algorithms can be deployed at the time of acquisition at UK Biobank centres to improve the image quality at source by alerting the radiographers early enough to allow reacquisition, e.g. with improved planning.

A limitation of this study was the different level of experience of the 13 observers, as identified by the inter-observer ICC that indicated the presence of small systematic differences between observers. To mitigate this problem, the final IQ scores were calculated as the average between observers weighted by their individual intra-observer ICC. Additionally, to correct major discrepancies between observers, IQ scores were reviewed by a panel in less than 7% of the scans. The sensitivity and specificity of the automated QC could be further improved and refined by adding features that can measure different aspects of the local image quality, e.g. vessel edge sharpness [17], and by re-training the RF model to classify IQ using these additional features.

This study used aortic cine data acquired in a single UK Biobank centre (in Cheadle). To move faster towards the goal of imaging 100,000 subjects, other 2 sites (in Newcastle and Reading) have been recently opened. The new centres use the same scanner (Siemens Aera) and CMR protocol to guarantee homogeneity of acquisition methods across the 3 centres, so we are confident that our automated method can be successfully applied to the entire UK Biobank dataset. Additionally, we have verified that it works on data acquired at 3 Tesla using different parameters (e.g. in-plane resolution) and, in principle, it should be applicable to any aortic cine acquired using the standard technique (i.e. ECG-gated bSSFP sequence). However, it should be further tested before performing automated analysis on aortic cine data from other centres using different sequences, scanner manufacturers and field strengths.

Finally, this study was concerned with the development and validation of automated methods for 1) aorta localization and 2) quality control. Measuring aortic distensibility requires the development and validation of additional automated algorithms to perform 3) frame-by-frame aortic lumen segmentation and 4) max-systolic and min-diastolic lumen area estimation. Part 3 and 4 constitute further planned work that will be addressed in a separate article.

## Conclusion

The proposed method for automated AA and PDA localization was extremely accurate and the automatically derived detection probabilities provided a robust mechanism to detect low quality scans for further human review. Applying the proposed localization and quality control techniques promises at least a ten-fold reduction in human involvement without sacrificing any accuracy. The proposed image quality control could also be used to streamline the image acquisition by alerting to the necessity of repeating acquisitions while patients are still in the scanner.

## Supporting information

S1 Fig User interface for the assessment of the image quality of aortic cine CMR. (TIF)

S2 Fig User interface for the detection of AA and PDA locations. Red circles indicate other candidate ROIs.
(TIF)

S1 File UK Biobank aortic analysis SOP. (PDF)

## Abbreviations

AA: Ascending Aorta
AUC: Area Under the Curve
bSSFP: balanced Steady State Free Precession
CHT: Circular Hough Transform
CMR: Cardiovascular Magnetic Resonance
DSC: Dice Similarity Coefficient
GT: Ground Truth
IQ: Image Quality
NA: Not Aorta
OOB: out-of-bag
PDA: Proximal Descending Aorta
PR: Precision Recall
RF: Random Forest
ROC: Receiver Operating Characteristic
