# Supplementary figures and images for: Automated localization and quality control of the aorta in cine CMR can significantly accelerate processing of the UK Biobank population data
Source: PLoS One. 2019 Feb 14;14(2):e0212272. doi: 10.1371/journal.pone.0212272 (PMC6375606; doi:10.1371/journal.pone.0212272)

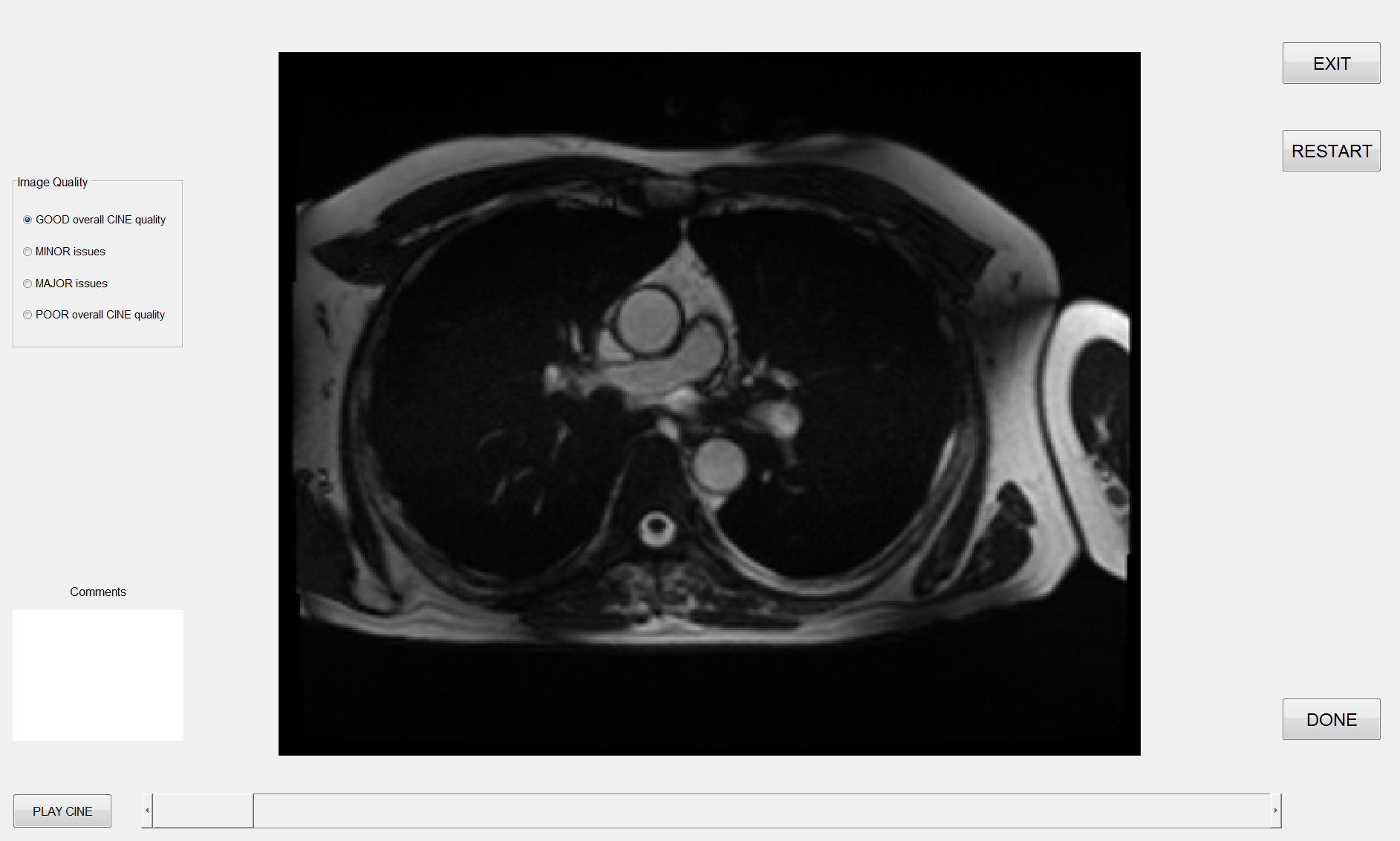

Supplement: S1 Fig — (TIF) [file pone.0212272.s001.tif]

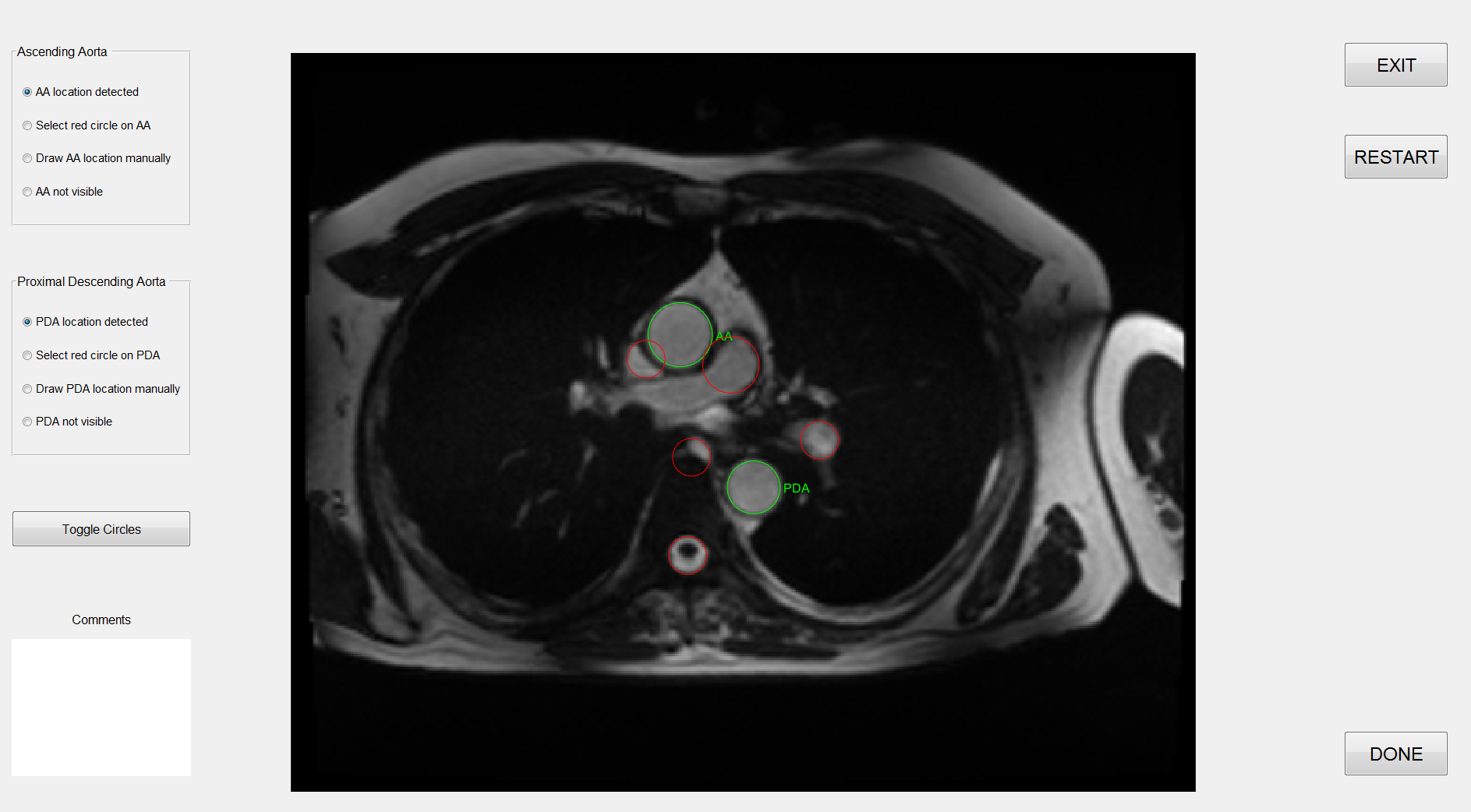

Supplement: S2 Fig — Red circles indicate other candidate ROIs. (TIF) [file pone.0212272.s002.tif]
