# Supplementary material for: Automated localization and quality control of the aorta in cine CMR can significantly accelerate processing of the UK Biobank population data
Source: PLoS One. 2019 Feb 14;14(2):e0212272. doi: 10.1371/journal.pone.0212272 (PMC6375606; doi:10.1371/journal.pone.0212272)
Supplement: S1 File — (PDF) [file pone.0212272.s003.pdf]

UKBB_Aortic_Analysis
Document references: OCMR_BHF_UK Biobank Imaging Pilot phase
Version Number: v.2.0
Creation Date: 11/10/2016
Effective Date: 11/10/2016
Title: Manual Validation of MRI Quality and Detection of Ascending and Proximal Descending Aorta.

Amendment Record

**Issue Number**	**Date**	**Change**	
		Signed:	Date:
Originator:	Name:	Signed:	Date:
Content approval:	Name:	Signed:	Date:
Quality Approval:	Name:	Signed:	Date:

Abbreviations:
OCMR – Oxford Centre for Magnetic Resonance
UKBB – UK BioBank project;
AA – ascending aorta;
PDA – proximal descending aorta;
QC – quality control;

Table of Contents

1. Introduction 3
2. System requirements and software installation 3
3. Dataset management 4
4. Validation Instructions 6
   Step 1: Evaluation and scoring of overall image quality 6
   Step 2: Identification of AA and PDA location 8
5. Examples of image quality 10
   A. Good quality images 10
   B. MINOR issues 10
   C. MAJOR issues 11
   D. Poor quality. 14
6. UI quick reference 16
7. Examples of keywords for free comments 17

1. Introduction

The current SOP describes how to carry out the validation of the results obtained from the computational method, developed by Dr L. Biasiolli (OCMR, The University of Oxford), to automatically detect ascending and proximal descending aorta (AA and PDA, respectively). The method was applied to the UK Biobank CMR pilot dataset. The validation process of the results of such method requires:

(1) Qualitative evaluation and scoring of general image quality.

(2) Identification of cases where either AA or PDA location was incorrectly detected by the automatic tool.

2. System requirements and software installation

The validation is carried out using a User Interface (UI) developed using Matlab (MathWorks) by Dr. Biasiolli, specifically for the purpose. A folder called *AoD_validation_yournamehere/* is provided for download to each observer, which contains all the software tools. The software is intended for use on 64 bit systems Windows only. A separate folder called *AoData/* is provided containing all the image dataset. This original folder is approximately 33GB, it is provided once to each observer/group of observers. *The folder can be stored on a hard disk drive or USB drive for portability. Keep the original folder for other observers and as a backup.*

1) **Download** the folder *AoD_validation_yournamehere/* into the same folder location containing *AoData/.* Rename the validation folder so that it contains the observer's name. Figure 1 shows how the folder should be renamed and located at the same level of *AoData/.* NOTE: If the observer plans on analysing his quota in batches, please, (a) have a separate folder for each batch; (2) specify the range in the folder name, for example, AoD_Luca_101_500, AoDLuca_1001_1500 etc...

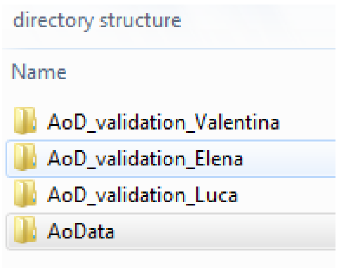

Figure 1 Folder renaming and location

2**) Install Matlab compiler**: inside the folder you will find **InstallMatlabRuntime.bat**. Click this file to make the software install the Matlab compiler. <u>NOTE. The installation takes a long while but needs to be done only once for each machine.</u>

3) You can now open the validation software by clicking on the **RunValidation.bat** file, as detailed in the following section.

Important additional information:

All the other sub-folders and files are not to be opened/edited, as they are used by the software to upload images and store results. To avoid modifying them, it is highly recommended to create a shortcut (soft link) to **RunValidation.bat** on the Desktop (or any preferred directory other than *Aod_validate_oxf_YourInitialsHere/*) and use that to open the software.

ATTENTION! Editing such files might accidentally cause the loss of your analysis!

3. Dataset management

To run validation software please use **RunValidation.bat**.

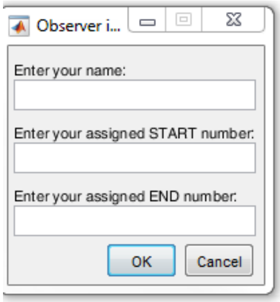

Figure 2. First access

NOTE: **The first time the software** is opened, it asks for the name of the observer, and the START and END index number that were assigned to the observer (Figure 2). The start and end index numbers will be provided to the observer according to the agreed time commitment. If you still do not know, please press cancel and wait for the indices to be provided.

Alternatively, you can test the software with any range, but then for the proper analysis you will have to start from a brand-new copy of the folder/software. This is why it is fundamental to always keep a backup of the original folder.

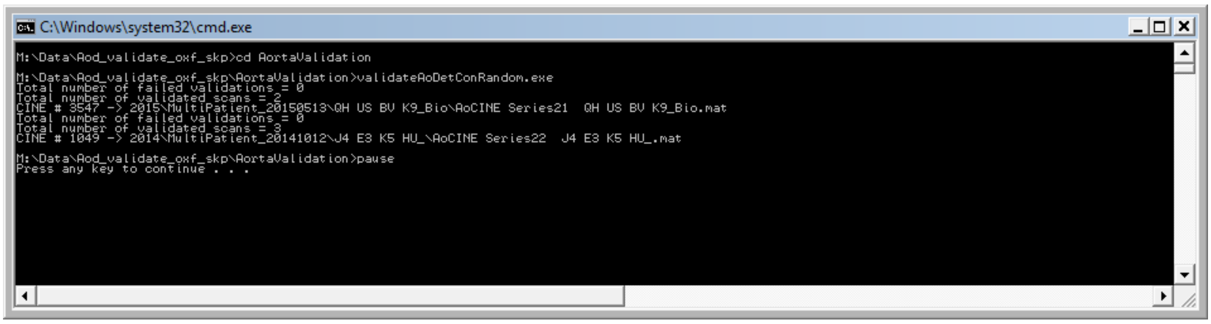

Figure 3. The log window in the background of the validation process. In case of problems, please remember to Alt+PrintScrn and save image in Paint, or use Top-left menu/Edit/Mark to record and paste the information text to be sent to us for troubleshooting.

Then proceed along instructions in section 4 for the proper validation session.

It is unlikely that all the assigned cases can be analysed in one session. On the contrary, observers are greatly encouraged to subdivide the workload in multiple sessions, to reduce the risk of errors.

After each session please click on **BackupDataAndForReturn.bat.** This backs up your work into the file *AoDResults_BackupReturnAfterFinished.rar*. This is also the file to be sent back to Oxford after all cases have been completed.

Please remember that, if for any reason problems arise during a session, there is always a log window open while the software is running. The log window is shown in Figure 3 and provides useful troubleshooting information. Please take a snapshot of the log window and send it back together with the description of the problem encountered.

4. Validation Instructions

In this section the details of the validation process are described as they are presented to the image analyst by the software for each case.

Step 1: Evaluation and scoring of overall image quality

As a new case is opened, the first window that appears is shown in Figure 4. This is the cine-MRI scan of AA and PDA. To evaluate the overall CINE image quality, use the **PLAY CINE** button and the scrollbar to go through all the 100 frames acquired during the cardiac cycle. The first frame is the diastolic phase. The systolic frame can approximately be identified by looking at the AA motion and increase in area.

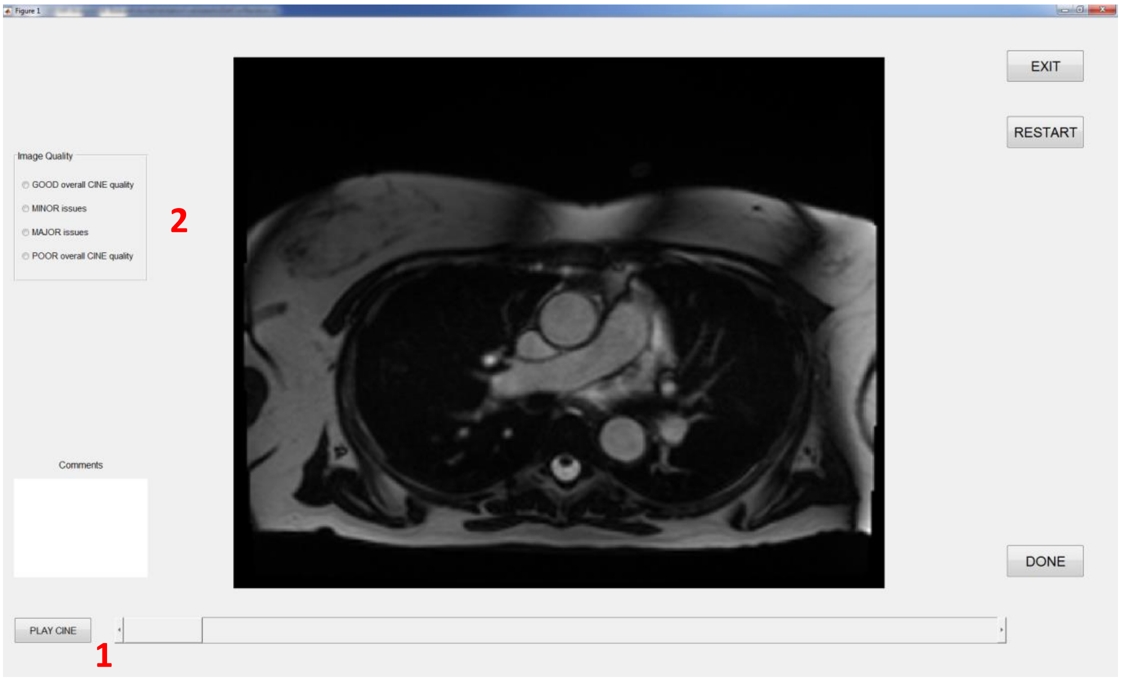

Figure 4. Image quality evaluation

1. Visually check the quality of the scan by playing the movie and/or by scrolling through the frames. In particular, check the quality of diastolic and systolic phase.
2. Score the overall quality of the scan. In section 5 the most common cases of sub-optimal image quality are described in order to guide the user in their decisions. The panel"Image Quality" on the upper left corner of the screen allows the observer to score the image as
   a. **Good overall CINE quality.** Tick this option if CINE images are clear with sharp AA and PDA lumen boundaries showing good image contrast throughout the cardiac cycle.
   b. **Minor issues.** Choose this option if the scan quality is sub-optimal due to minor blurring or ghosting but AA and PDA lumen boundaries can still be easily and consistently identified (see examples in section 5).
   c. **Major issues.** Choose this option if the CINE quality is corrupted by major artefacts or if the slice location is not optimal. In this case AA and PDA lumen boundaries can be affected by major artefacts (see examples in section 5), but still be visible for most of the cardiac cycle. This option is valid also if AA is not visible but PDA still is (or vice versa), because the analysis of AA and PDA are independent.
   d. **Poor quality**. Choose this option if the quality of the scan is generally very poor and both AA and PDA lumen boundaries are severely corrupted or if the slice location is completely incorrect.
3. If **b**, **c** or **d** is selected, then a second panel appears to specify the reason for such choice (see Figure 5). The options are:
   - Some images are corrupted;
   - Most images are corrupted;
   - Incorrect slice location.

   The first and second options are mutually exclusive. The third option can be chosen alone or in combination with either the first or the second. In other words, a scan can have corrupted images and wrong slice location at the same time.
4. It is possible to add free text comments with additional information on general image quality (please refer to section 7 for keywords).

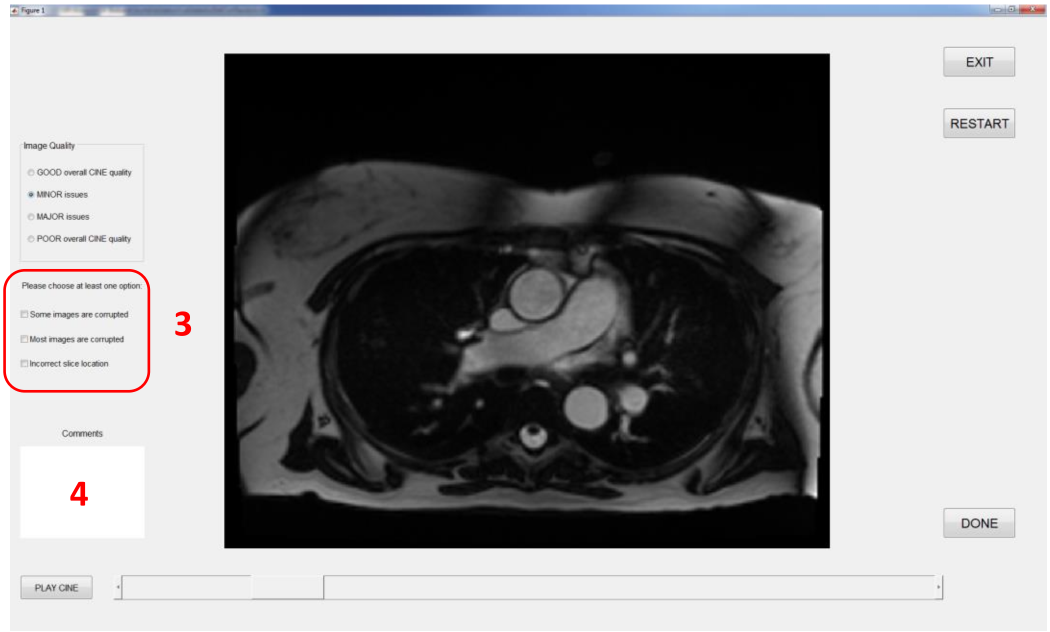

Figure 5. Image quality evaluation (continued)

If the user is happy with the validation of the general image quality, he/she can click on **DONE**, in the lower right corner to proceed to the next part of the validation process.

RESTART button will reset anything that has been clicked or written in this window, allowing to start afresh on this case.

EXIT button will close the program. **ATTENTION!** Anything done at this stage for the current case will be lost. If the observer needs to exit the program, please do it here before performing any quality control to avoid losing any unsaved data.

Step 2: Identification of AA and PDA location

After clicking DONE in the previous window, a second window will appear showing the diastolic frame as before, but this time with overlaid the results of the automatic aortic detection tool. The results consist of a set of green and red circles, as shown in Figure 6. The automatic detection identifies a number of circular structures in the frame, those shown in red. It then identifies AA and PDA circles based on a complex combination of parameters. Once identified, it shows the corresponding circles in green.

The goal of the analyst is to tell whether AA and PDA were correctly identified or not. Keeping in mind that the analysis of AA and PDA is independent, and for this reason there are two separate panels showing the same options in both cases, the following instructions explain the case of AA, but they are valid also when assessing PDA.

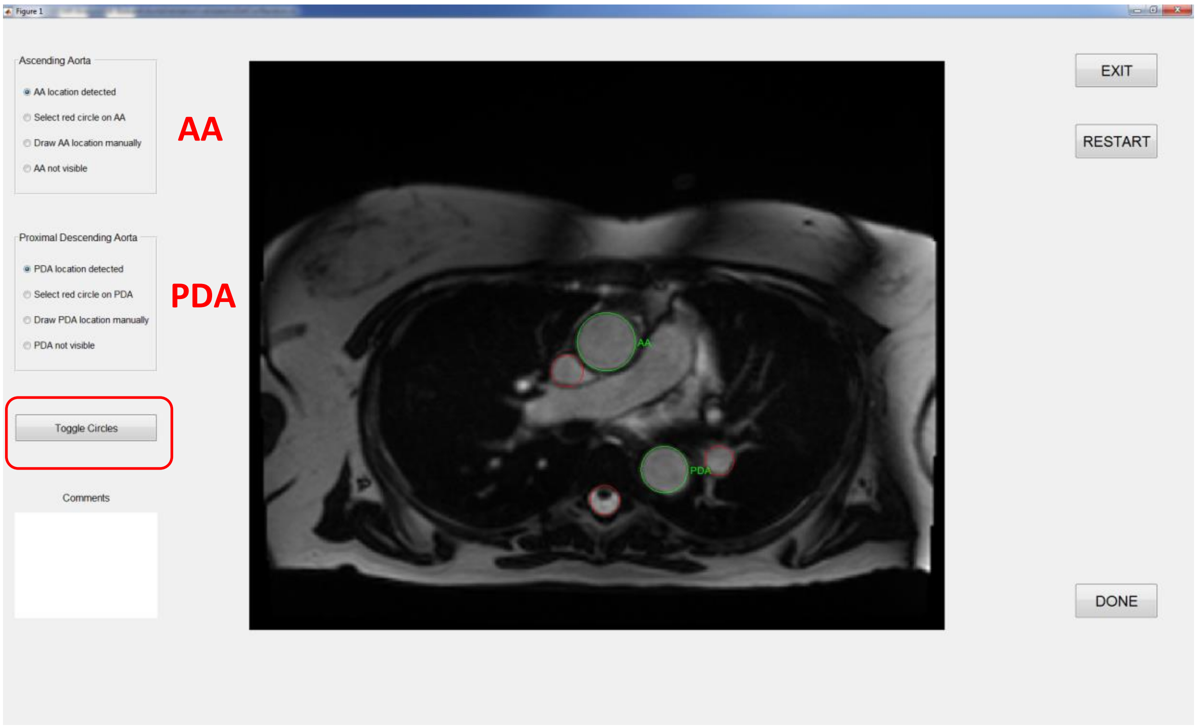

Figure 6. Identification of AA and PDA location

Case 1. AA location detected. A green circle identifies the AA location. At this stage the lumen contour is not important, as long as the circle indicates the correct anatomical structure. There will be other red circles, but they can just be ignored.

Case 2. Select red circle on AA. The green circle is not present or it is assigned to the wrong anatomical structure, but the correct AA location is identified by a red circle. In this case the observer will select the correct red circle by double-clicking inside it. (Single click does not work).

Case 3. Draw AA location manually. The green circle is not present or it is assigned to the wrong anatomical structure, and the correct AA location is not identified by a red circle either. In this case the observer will draw the AA lumen boundaries manually by clicking few points around the lumen boundary.

Case 4. AA not visible. The aorta is not visible in the image.

The button **Toggle Circles** hides/shows all the green and red circles.

Click **DONE** to finish the identification of AA and PDA location and open the next part of the validation process.

5. Examples of image quality

In this section a few examples are shown in order to provide the user with a guide to classify image quality.

A. Good quality images

Both AA and PDA circles are clearly visible and no artefact is affecting them (Figure 7). The slice location is correctly cutting through the middle of the pulmonary artery (PA). AA and PDA are circular and the pulmonary artery (PA) is visible through the image in a nice streamline shape separating the two regions of AA and PDA (Figure 8).

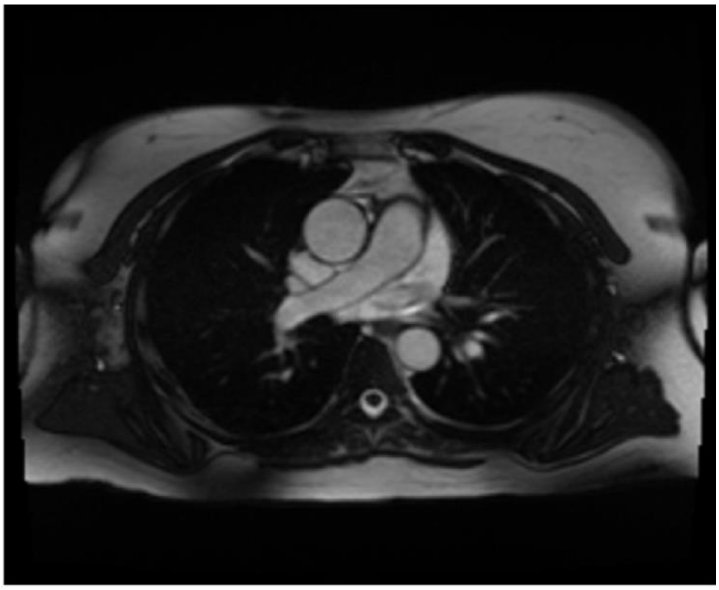

Figure 7. Good quality scan

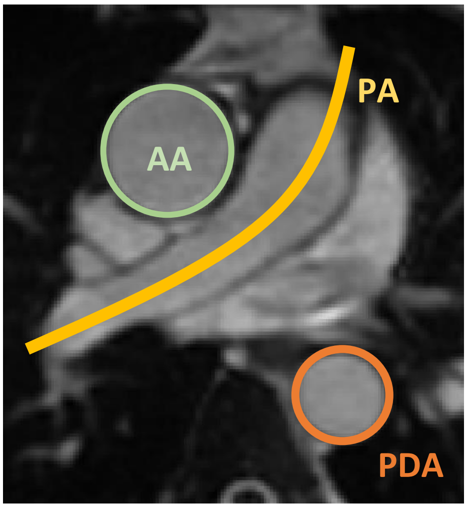

Figure 8. Zooming in

B. MINOR issues

The scan quality is sub-optimal due to minor blurring or ghosting but AA and PDA lumen boundaries can still be easily and consistently identified. See, for example, Figure 10 and Figure 9.

The validation does not require specifying the nature of the artefact, only the extent to which it affects the image quality.

An example of a minor issue caused by sub-optimal slice location is given in Figure 11.

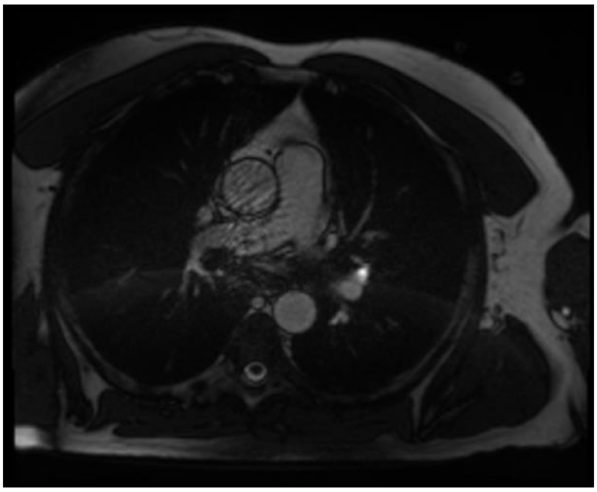

Figure 10. Minor issues: ghosting-- the lumen boundaries are blurred but both AA and PDA boundaries are still clearly visible.

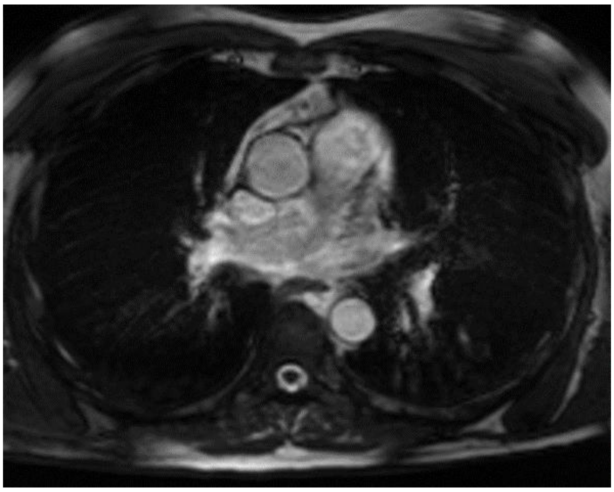

Figure 9. Minor issues: blurring - image affected by a minor ghosting artefact where both AA and PDA lumen boundaries remain relatively sharp and clearly visible.

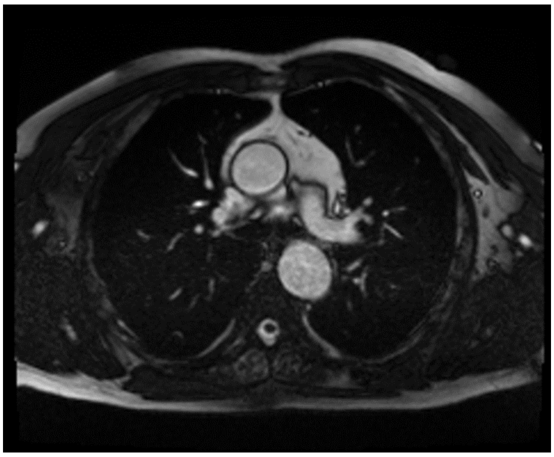

Figure 11. Minor issues: slice location -- pulmonary artery is not visible and the PDA has an ellipsoidal shape. However, by looking at the CINE movie, it is possible to check that in this case PDA is moving/dilating smoothly, therefore it may still be used for distensibility analysis.

C. MAJOR issues

Tick this option if the presence of strong artefacts affects the quality of AA and/or PDA lumen boundaries, but they are still visible for most of the cardiac cycle, or if the slice location is sub-optimal. You can also tick this option if AA image quality is corrupted but PDA is still clearly visible (or vice versa), because the analysis of AA and PDA are independent.

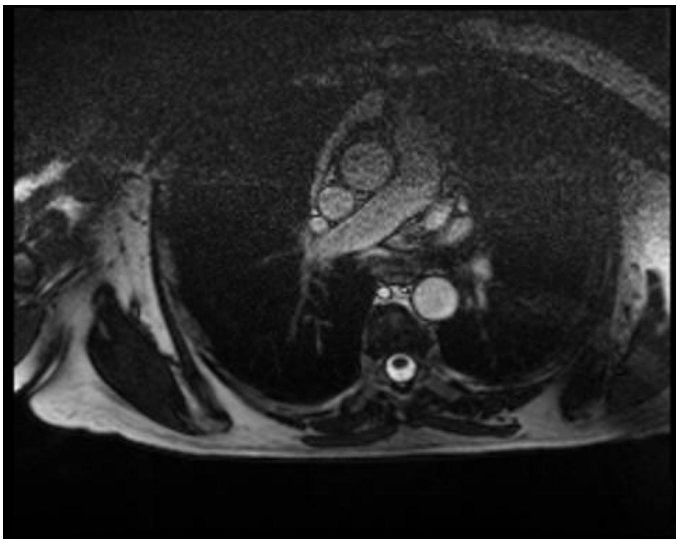

Figure 12. Major issues: low SNR -- AA is severely affected by low SNR on all images, but PDA is clearly visible

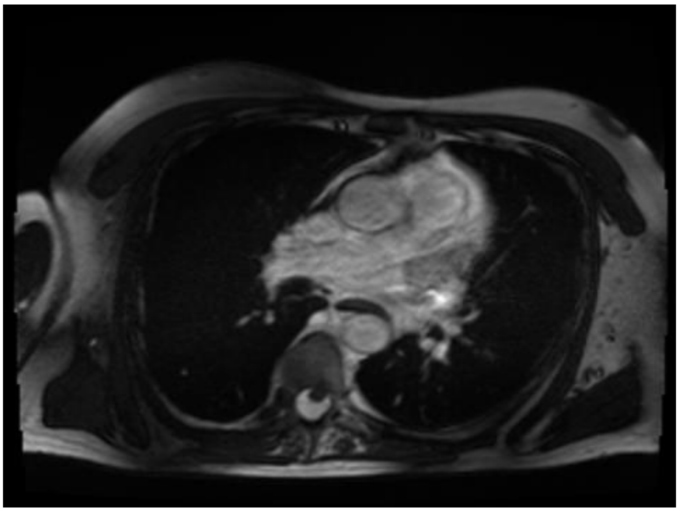

Figure 13. Major issues: blurring -- severe blurring on both AA and PDA lasting for few images, after which the lumen boundaries return to be more visible

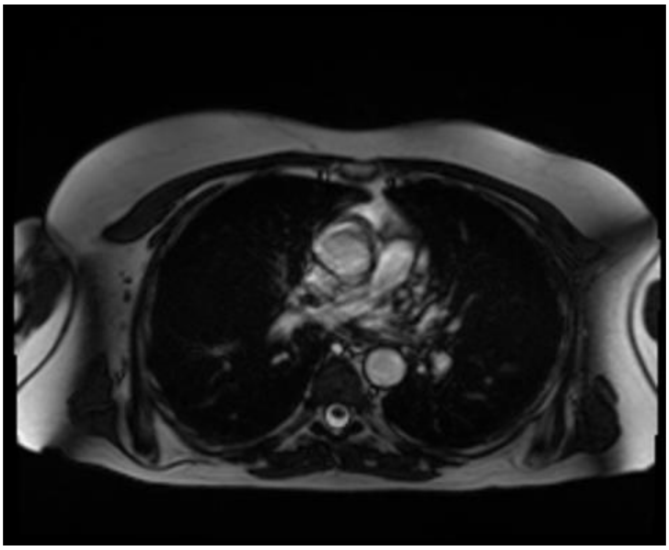

Figure 14. Major issues: AA ghosting -- AA affected by ghosting for several images, but PDA still clearly visible

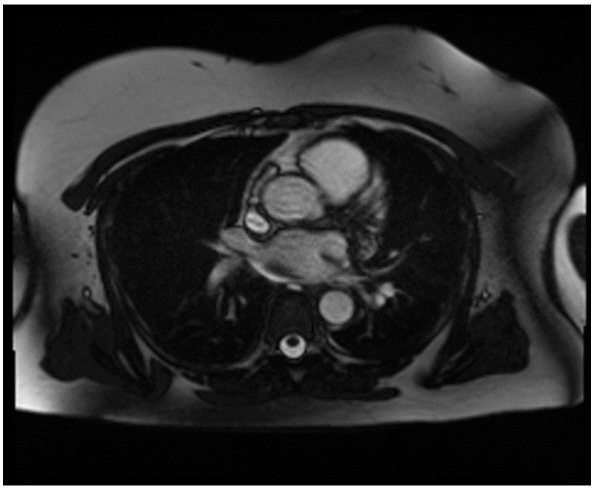

Figure 16. Major issues: incorrect slice location -- scan with incorrect slice location, cutting below the pulmonary artery. PDA is still visible and has a circular shape, so its distensibility can still be analysed

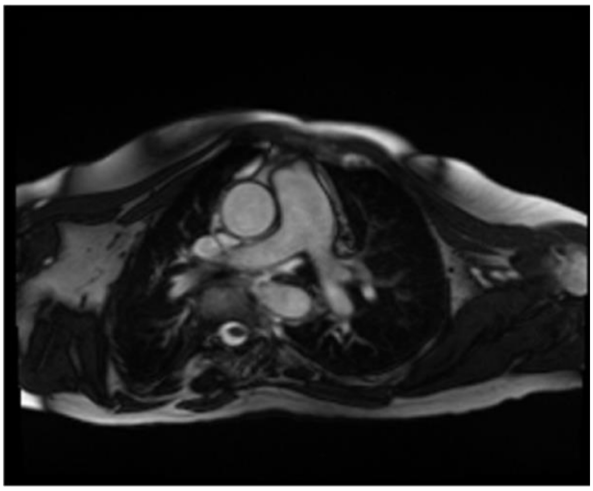

Figure 15. Major issues: oblique slice location on PDA -- the image slice cuts through the PDA obliquely, causing an irregular and non-circular shape, which does not allow distensibility analysis

D. Poor quality.

Choose this option if the quality of the scan is generally very poor and both AA and PDA lumen boundaries are severely corrupted, or if the slice location is completely incorrect.

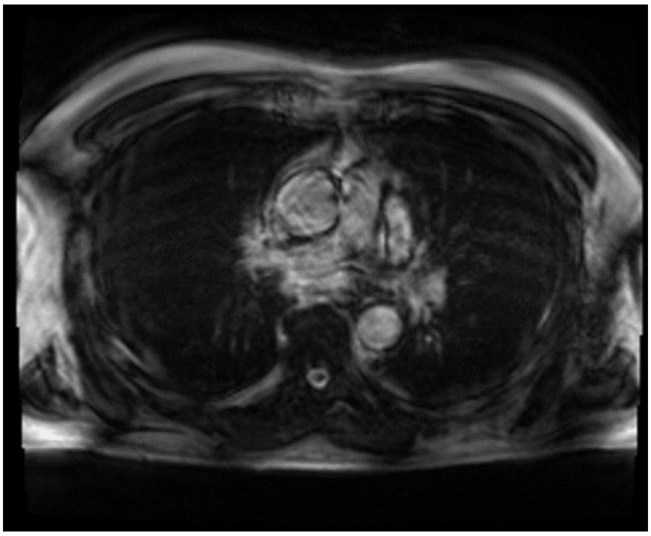

Figure 17. Poor quality due to motion artefacts -- CINE series corrupted by motion artefacts throughout the entire cardiac cycle

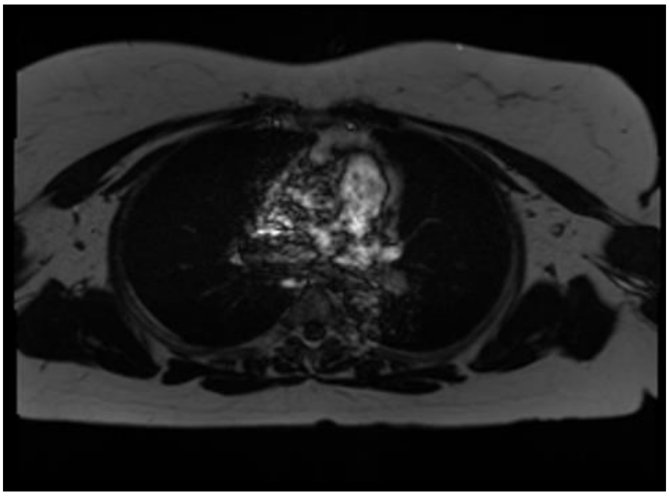

Figure 18. Poor quality due to off-resonance artefacts -- severe off-resonance artefacts that affect both AA and PDA for several images during the cardiac cycle.

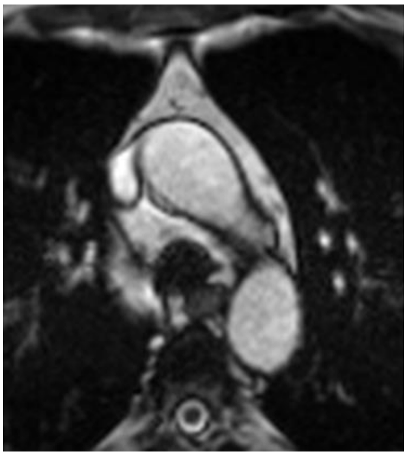

Figure 19. Poor quality due to slice location -- incorrect slice location. The slice was positioned too high above the pulmonary artery and cuts the aorta approximately at the level of the aortic arch.

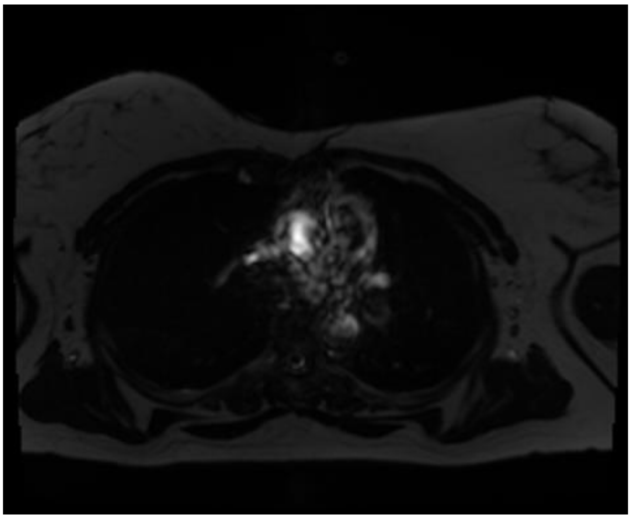

Figure 20. Poor quality due to off-resonance artefacts -- severe off-resonance artefacts that affect both AA and PDA for several images during the cardiac cycle.

6. UI quick reference

Step 1: Evaluation and scoring of image quality.

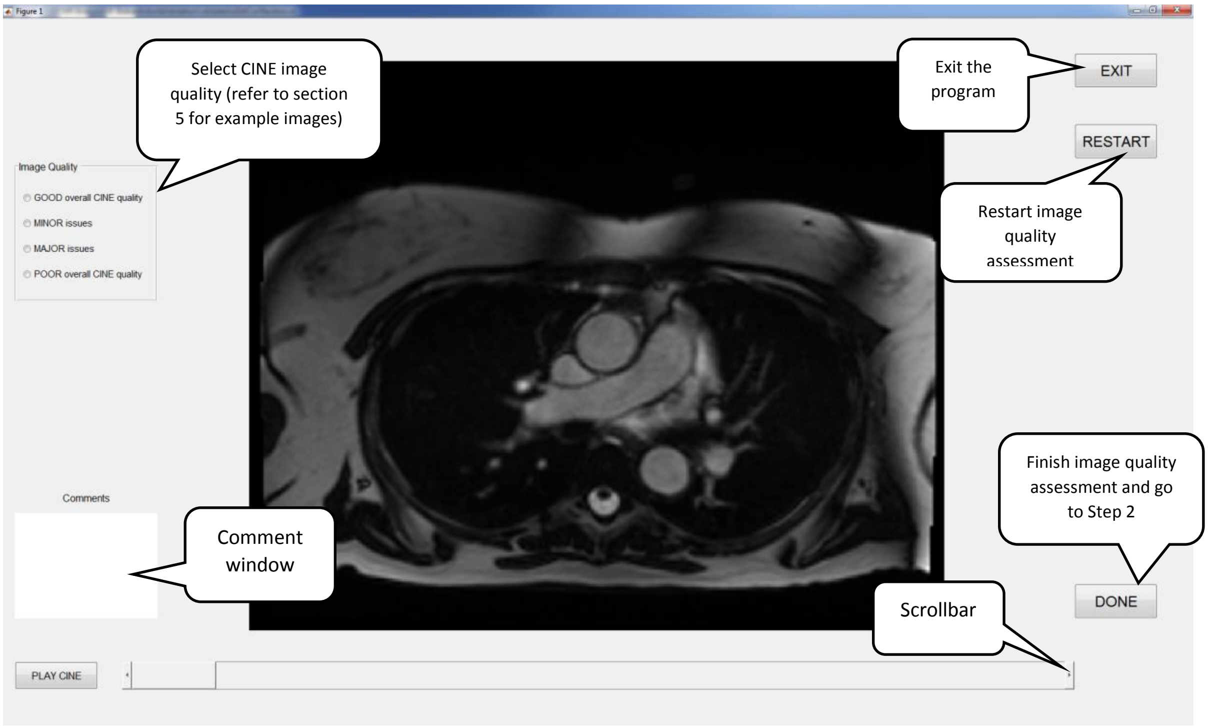

PLAY CINE button and **scrollbar** to check all the frames acquired during the cardiac cycle.

Select **image quality** as one of the four main categories + free text **comments** with extra information about general image quality can also be added (see list of keywords?).

DONE to go to the next validation step.

RESTART to reset anything that has been clicked or written in this window, allowing to start afresh on this case. Click **EXIT** to close the program - . **ATTENTION!** The current case will be need to be re-analysed.

Step 2: Identification of AA and PDA location

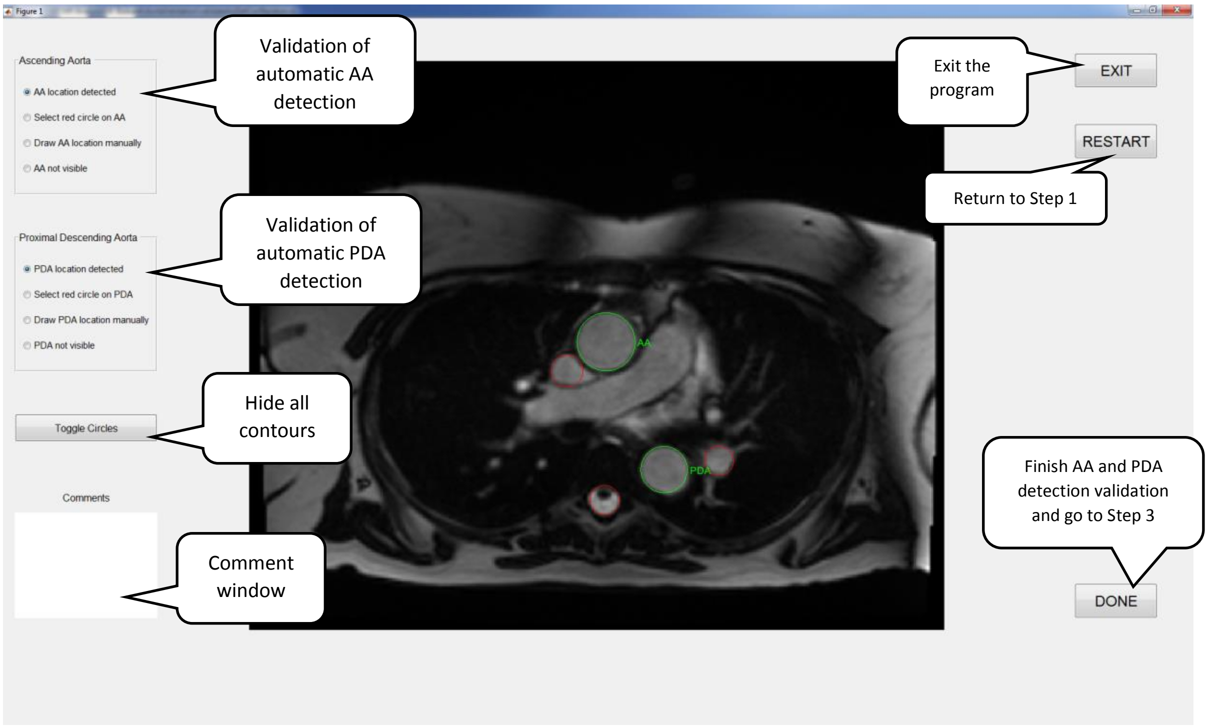

7. Examples of keywords for free comments

- Incorrect slice location. The plane is too oblique.

- Difficult to identify systole or diastole due to stiffness.

- Artefacts, affect AA/PDA.

- AA/PDA not analysable due to artefacts.

- AA/PDA wrongly identified. Manual drawing.

- Automatic contouring failed during artefacts. Manual drawing.

- AA/PDA not visible due to artefacts.

- Poor quality overall, not analysable.
